# Linguistic spin in randomized controlled trials about age-related macular degeneration

**DOI:** 10.3389/fepid.2022.961996

**Published:** 2022-10-31

**Authors:** Nienke Veldhuis, Myrthe A. Nuijts, Luka Isphording, Felicia V. Y. L. Lee-Kong, Saskia M. Imhof, Inge Stegeman

**Affiliations:** ^1^Faculty of Medicine, Utrecht University, Utrecht, Netherlands; ^2^Department of Ophthalmology, University Medical Center Utrecht, Utrecht, Netherlands; ^3^Department of Otorhinolaryngology and Head and Neck Surgery, University Medical Center Utrecht, Utrecht, Netherlands; ^4^Brain Center Rudolf Magnus, University Medical Center Utrecht, Utrecht, Netherlands

**Keywords:** linguistic spin, age-related macular degeneration (AMD), randomized controlled trial, ophthalmology, overinterpretation

## Abstract

**Objective:**

To evaluate the prevalence, type and extent of linguistic spin in randomized controlled trials (RCTs) exploring interventions in patients with age-related macular degeneration (AMD), as well as to investigate whether study variables were correlated with linguistic spin.

**Study design and setting:**

PubMed was searched from 2011 to 2020 to identify RCTs including patients with AMD. Two authors independently assessed a total of 96 RCTs. Linear regression analyses were performed to investigate whether linguistic spin was correlated with predefined study variables.

**Results:**

Linguistic spin was found in 61 of 96 abstracts (63.5%) and in 90 of 96 main texts (93.8%). Use of words pointing out the beneficial effect of a treatment and the use of ‘(statistically) significant/significance' without reporting a *P*-value or a 95% confidence interval (CI) were the most frequently identified categories of linguistic spin. Sample size was significantly correlated with the total linguistic spin score (95% CI 0.38–5.23, *P* = 0.02).

**Conclusion:**

A high prevalence and extent of linguistic spin in RCTs about AMD was found. We highlighted the importance of objective reporting and awareness of linguistic spin among ophthalmologists and other readers.

## Introduction

Evidence-based medicine (EBM) depends on the best available scientific evidence, clinical knowledge and the needs, values and desires of the patient (1). Accurate and reliable research is crucial for EBM. Randomized Controlled Trials (RCTs) are considered as the most reliable study design for assessing the safety and efficacy of new treatments. Because clinicians' decisions often rely on RCTs, adequate and reliable reporting is one of the challenges in biomedical research (2).

Spin, reporting that distorts or misrepresents study results to make the interventions look favorable, may result in suboptimal or even harmful treatment decisions (3–7). Spin can be the consequence of either unconscious or conscious behavior to interest readers: it highlights the beneficial effect of the intervention and or suppresses negative results (3, 5, 8). In the past decade, there has been a focus on the quality of biomedical research (9–12). Regulations and guidance have been offered to researchers to help them with conducting methodological sound research and adequate reporting (13). While these guidelines are developed to help researchers, they have a high level of autonomy in what they write and how they present their results. Regrettably, to date, the reporting of scientific reports remains suboptimal (5, 6, 14–16).

There are various types of spin that could be distinguished in scientific reports. Lazarus et al. (4) classified spin into the following three categories: “misleading reporting,” “misleading interpretation” and “inadequate extrapolation of the results.” Each of the mentioned categories identifies multiple strategies of spin (4). One of these strategies includes linguistic spin, which is defined as the (un)conscious use of language to convince readers of the beneficial effect of the intervention. Examples of linguistic spin include “a positive trend,” “highly significant” or “excellent results” (7, 17, 18). Linguistic spin is known as one of the most used spin strategies in several domains of biomedical research (4). Although some researchers have argued that linguistic spin is to some extent inherent to scientific writing (8, 15), previous research investigating other categories of spin demonstrated that it can distort the reader's interpretation of study results (7, 17, 18).

We decided to assess linguistic spin in the field of ophthalmology. At this moment, age-related macular degeneration (AMD) is a common disease in ophthalmology and is the main cause of irreversible blindness in people aged above 50 years in developed countries. Nevertheless, no curable therapy is available for AMD (19–22). More accurate reporting might help in finding an effective treatment for AMD. Therefore, in this study we assess the prevalence, type and extent of linguistic spin in abstracts and main texts of RCTs about interventions in patients diagnosed with AMD, and investigate whether study variables are correlated with linguistic spin.

## Methods

### Information sources and search strategy

PubMed was searched on April 12, 2020, to identify all eligible studies. The database was searched for the following search terms in title or abstract, together with their synonyms and or abbreviations: “macular degeneration,” “maculopathy,” “macular dystrophy” and “AMD.” To identify studies with the study design of interest we used the Cochrane collaboration sensitivity-maximizing filter for RCTs (23). The full search strategy is presented in Supplementary Table A. No publication status restrictions were used. All studies identified were managed using Rayyan QCRI (24).

### Study selection

Two authors (NV and LI) independently conducted title and abstract screening of studies identified from the electronic search. Potentially relevant papers were read independently in full-text to assess their eligibility for inclusion. Any disagreements were resolved by discussion and, if required, the article was discussed with a third author.

### Eligibility criteria

Title/abstract and full-text screening were performed based on predefined in- and exclusion criteria. We included primary reports of RCTs investigating a treatment in patients with AMD. We defined RCTs as prospective studies assessing a clinical intervention in human subjects assigned, at random, to one of multiple study groups. Randomized controlled trials with more than two study arms or crossover trials were included as well. We excluded cost-effectiveness studies, reports of diagnostic accuracy, equivalence or non-inferiority trials, interim analyses, cluster trials, factorial or split-body designs, phase 1 and 2 trials, pilot studies, feasibility studies and extension studies. Reports not available in English or Dutch were also excluded.

### Outcome measures

The primary outcome measures of this study were the prevalence, type and extent of linguistic spin in RCTs about patients with AMD. The secondary outcome measure of our study was to investigate whether predefined study variables (i.e., study group, journal impact factor, journal endorsing CONSORT guidelines, international collaboration, number of citations, number of treatment arms, sample size and funding) were correlated with linguistic spin.

### Data extraction

Data extraction was carried out by two authors (NV and LI) independently. Any inconsistencies were resolved through discussion. The following study characteristics were extracted: first author, title, journal, year of publication, study group, journal impact factor, journal endorsing CONSORT guidelines, international collaboration, number of citations, number of treatment arms, sample size and funding.

The CONSORT Statement website was used to define whether or not a journal endorsed CONSORT guidelines (25). If the study was performed in centers located in two or more countries, this was indicated as an international collaboration. We used Scopus to determine the number of citations for each study individually (26) and InCites to identify the journal impact factor (27). Four study groups were defined: studies with a statistically significant result for the primary outcome (study group 1), studies with a statistically non-significant result for the primary outcome (study group 2), studies in which a primary outcome was not clearly specified (study group 3) and studies in which the significance of the primary outcome was not reported (study group 4).

### Linguistic spin assessment

Linguistic spin was defined as the (un)conscious use of language to convince readers of the beneficial effect of the intervention and or to suppress negative results (28–30). We contacted various authors with expertise on (linguistic) spin by e-mail, aiming to find a database of linguistic spin examples. However, no such database appeared to be available and, therefore, we decided to define linguistic spin categories ourselves. The following seven categories of linguistic spin were defined: (1) Use of words to reject or explain non-statistically significant results; (2) Use of words to claim comparable effectiveness or equivalence despite a *P* > 0.05; (3) Use of words to point out the beneficial effect of the treatment investigated (e.g. “excellent” results); (4) Use of “(statistically) significant/significance” without reporting a *P*-value or a 95% confidence interval (CI) for results showing a beneficial effect of the treatment investigated; (5) Particular focus on results with statistical significance in abstract and or main text (i.e., only pointing out the statistically significant results); (6) Inconsistency in the significance of the same study results between different sections of the article and (7) Other forms of linguistic spin. An overview of the seven linguistic spin categories together with examples is provided in Supplementary Table B.

Two authors (NV and FLK) independently assessed linguistic spin in the included RCTs. Categories of linguistic spin were applied to different sections—i.e., abstract results and conclusion; main-text results, discussion and conclusion—except for linguistic spin category 6. The total linguistic spin score was determined by summarizing the number of linguistic spin examples in the article for each study individually. This was used to examine the extent of linguistic spin into more detail, but we do not suggest to use this as a scoring system for linguistic spin.

### Statistical analysis

Categorical data are presented as frequencies with percentage (%), continuous data are presented as mean with standard deviation (±SD) (normally distributed data), or as median with ranges (not normally distributed data). Univariate linear regression analysis was used to assess whether linguistic spin was correlated with predefined study variables. A *P* < 0.05 was considered statistically significant. We analyzed the collected data using Statistical Package for the Social Sciences version 25.0.0.2 (SPSS Inc, Chicago, Illinois, USA).

## Results

### Study selection

**Figure 1:**
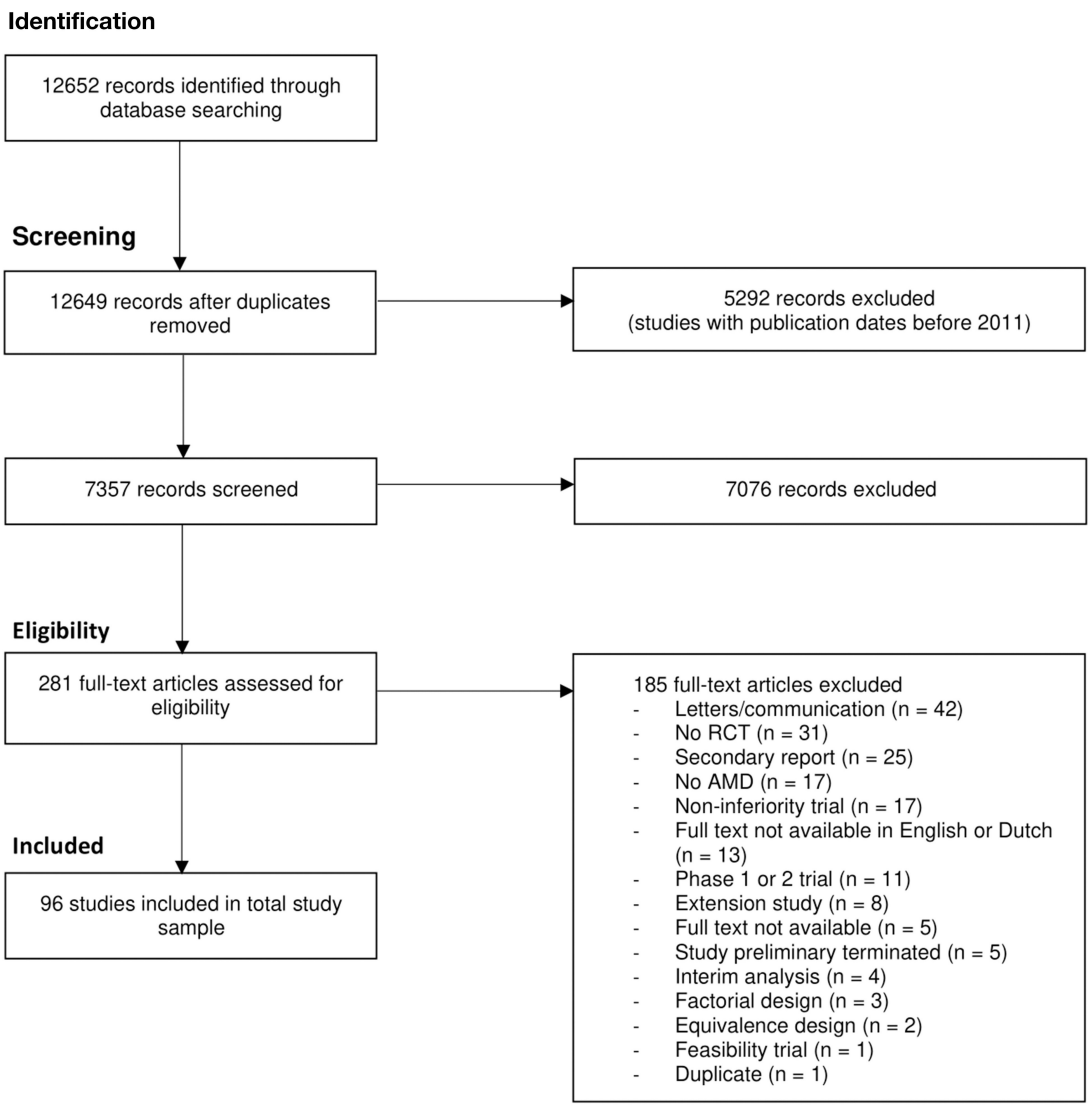
PRISMA flow chart for identification and selection of studies. AMD, age-related macular deeneration; RCT, randomized controlled trial.

We retrieved 12.652 records through our literature search in PubMed. After removal of duplicates, title and abstract screening was performed on 7357 records. A total of 281 full-text articles were assessed. Finally, we included 96 RCTs about interventions in patients with AMD. The study identification, selection process and reasons for exclusion are shown in Figure 1.

### General characteristics of included studies

The median sample size of the included studies was 70 patients (75 eyes), ranging from 14 to 1,520 patients (16–2586 eyes). The median journal impact factor was 2.81 (range 0.12–9.67) and the median number of citations was 12.5 (range 0–122). Twenty-seven studies (23.7%) reported to have adhered to the CONSORT guidelines. Five studies (4.4%) were international collaborations. The median number of treatment arms of the included studies was 2 (range 2–4). In 22 studies (19.3%) a statistically significant primary outcome was identified, in 30 studies (26.3%) a non-statistically significant primary outcome, in 42 studies (36.8%) a primary outcome was not clearly specified and in two studies (1.8%) authors did not refer to statistical significance for the primary outcome. Funding was reported in 76 studies (79.2%), in 49 studies (43%) the obtained funding was non-profit, in 18 studies (15.8%) for-profit, in seven studies (6.1%) both non-profit and for-profit and in two studies (1.8%) funding was not clearly described. A summary of the study characteristics is shown in Table 1 and a detailed overview of study characteristics for each included study is presented in Supplementary Table C.

**Table 1:** General study characteristics of the included studies (*N* = 96).

**Study characteristic**	***N* (%) or median (ranges)**
Total number of studies included	96
Sample size	
Patients	70 (14–1520)
Eyes	75 (16–2586)
Journal impact factor* (*N* = 95)	2.81 (0.12–9.67)
Number of citations* (*N* = 92)	12.5 (0–122)
Study group	
1. Statistically sign. primary outcome	22 (19.3)
2. No statistically sign. primary outcome	30 (26.3)
3. Primary outcome not clearly specified	42 (36.8)
4. No reference to statistical sign. for primary outcome	2 (1.8)
Endorsing CONSORT guidelines	27 (23.7)
International collaboration	5 (4.4)
Number of treatment arms	2 (2–4)
Funding	
Non-profit	49 (43)
For-profit	18 (15.8)
Both	7 (6.1)
Unclear	2 (1.8)
Not reported	20 (17.5)

*If the total number of studies differs from 96 studies this is indicated. Sign., significant or significance.

### Prevalence of linguistic spin in abstracts and main texts

A detailed overview of linguistic spin examples in the included RCTs is provided in Supplementary Table D. Linguistic spin was identified in 61 of the 96 (63.5%) abstracts. The prevalence of the seven linguistic spin categories for each section of the text is shown in Table 2. The most common category of linguistic spin in abstracts was linguistic spin category 4 [“Use of ‘(statistically) significant/significance' without reporting a *P*-value or a 95% CI for results showing a beneficial effect of the treatment investigated”]. This category was present in the results section of 30 (31.3%) abstracts and the conclusion section of 16 (16.7%) abstracts.

**Table 2:** Classification of linguistic spin in abstracts and main texts of RCTs about AMD.

**Linguistic spin categories**		**Abstract (N** = **96)**		**Main text (N** = **96)**			**Total**
		**Results**	**Conclusion**	**Results**	**Discussion**	**Conclusion**	
		N*(%)/N**	N*(%)/N**	N*(%)/N**	N*(%)/N**	N*(%)/N**	N*(%)/N**
1	Use of words to reject or explain non-statistically significant results	2 (2.1)/2	0 (0)/0	12 (12.5)/15	12 (12.5)/12	0 (0)/0	26 (5.4)/19
2	Use of words to claim comparable effectiveness or equivalence despite *P* > 0.05	3 (3.1)/5	0 (0)/0	6 (6.3)/6	1 (1.0)/1	2 (2.1)/2	12 (2.5)/14
3	Use of words to point out the beneficial effect of the treatment investigated	11 (11.4)/14	12 (12.5)/15	38 (39.6)/58	50 (52)/79	29 (30.2)/34	140 (29.2)/200
4	Use of ‘(statistically) significant/significance' without reporting a *P*-value or a 95% CI for results showing a beneficial effect of the treatment investigated	30 (31.3)/45	16 (16.7)/16	33 (34.4)/63	54 (56.3)/145	15 (15.6)/18	69 (14.4)/287
5	Particular focus on results with statistical significance in abstract and or main text	6 (6.3)/6	1 (1.0)/1	0 (0)/0	0 (0)/0	0 (0)/0	7 (1.5)/7
6	Inconsistency in the significance of the same study results between different sections of the article	NA	NA	NA	NA	NA	5 (1.0)/5
7	Other	1 (1.0)/1	5 (5.2)/5	5 (5.2)/5	33 (34.4)/45	7 (7.3)/7	51 (10.6)/63

*Number of articles in which a linguistic spin category is found. **Total number of linguistic spin examples of a specific category for all included articles (one category of spin can be scored several times in one article). NA, not applicable.

Linguistic spin was identified in 90 of the 96 (93.8%) main texts. For main texts, the most frequently identified linguistic spin category was category 3 (“Use of words to point out the beneficial effect of the treatment investigated”), which was present in the results, discussion and conclusion section of respectively 38 (39.6%), 50 (52%) and 29 (30.2%) main texts. In five of the 96 included RCTs (1.0%) we identified linguistic spin category 6 (“Inconsistency in the significance of the same study results between different sections of the article”).

### Extent of linguistic spin in abstracts and main texts

Ten studies had linguistic spin in all sections of the abstract (10.4%) and 30 studies had linguistic spin in all sections of the main text (31.3%). Linguistic spin was identified in the conclusion section only in 17 abstracts (17.8%) and three main texts (3.2%) (Table 3). The mean total linguistic spin score was 6 (range 0–26). In five studies (5.2%) no linguistic spin was identified in both the abstract and main text of the article.

**Table 3:** The extent of linguistic spin in abstracts and main texts of RCTs about AMD.

**Extent of linguistic spin**	**Abstract (N = 96)**	**Main text (N = 96)**
	**N (%)**	**N (%)**
None	35 (36.5)	6 (6.3)
1 section, other than conclusion section	34 (35.4)	87 (90.6)
Conclusion section only	17 (17.8)	3 (3.2)
Discussion section only	NA	16 (16.7)
2 sections	NA	37 (38.5)
All sections	10 (10.4)	30 (31.3)

NA, not applicable.

### Correlation between study variables and total linguistic spin score

Sample size was found to be positively correlated with the total linguistic spin score (patients: B = 2.80, 95% CI 0.38–5.23, *P* = 0.02; eyes: B = 2.71, 95% CI 0.25–5.13, *P* = 0.03). Other study variables were not statistically significantly correlated with the total linguistic spin score (Table 4). Due to the small sample size of study group 4 (*N* = 2), only descriptive statistics were reported for this study group.

**Table 4:** Study variables correlated with total linguistic spin score in RCTs about AMD.

**Study variable**	**Unstandardized regression coefficient (B) (95% CI)**	P*
**Study group**		
1. Statistically sign. primary outcome	−0.51 (−2.89 to 1.88)	0.67
2. No statistically sign. primary outcome	0.32 (−1.84 to 2.49)	0.77
3. Primary outcome not clearly specified	Ref	Ref
4. No reference to statistical sign. for primary outcome**	NA	NA
Journal impact factor	−0.23 (−0.70 to 0.25)	0.34
Journal endorsing CONSORT	0.60 (−1.44 to 2.63)	0.56
International collaboration	1.99 (−2.12 to 6.10)	0.34
Number of citations	1.41 (−0.42 to 3.24)	0.13
Number of treatment arms	0.56 (−1.06 to 2.19)	0.49
Sample size		
Patients	2.80 (0.38 to 5.23)	0.02*
Eyes	2.71 (0.25 to 5.13)	0.03*
Funding		
Non profit	Ref	Ref
For-profit	−0.38 (−2.86 to 2.09)	0.76
Both	2.10 (−1.53 to 5.73)	0.25
Unclear	−1.33 (−7.80 to 5.15)	0.69
Not reported	0.15 (−0.91 to 3.86)	0.22

*P < 0.05 was considered statistically significant. **Only descriptive statistics were reported for this study group. Sign., significant or significance; Ref, reference group; NA, not applicable.

## Discussion

This study was designed to assess the prevalence, type and extent of linguistic spin in abstracts and main texts of RCTs about interventions in patients diagnosed with AMD. In addition, we aimed to investigate whether predefined study variables were correlated with linguistic spin.

Overall, we found linguistic spin in 63.5% of the abstracts and 93.8% of the main texts. Authors most commonly used words to point out the beneficial effect of a treatment and or reported “(statistically) significant/significance” results without reporting a *P*-value or a 95% CI showing a beneficial effect of the treatment. A statistically significant correlation (*P* < 0.05) was observed between the total linguistic spin score and sample size.

In the present study, higher percentages of linguistic spin were found than in previous studies investigating linguistic spin in other fields. Chiu et al. (31) systematically reviewed the prevalence of spin in general in published biomedical literature and reported a median of 56.8% (9.7–83.6%) spin in abstracts and a median of 56.5% (18.8–100%) spin in main texts of (non-) randomized controlled trials. Two previous studies primarily assessed linguistic spin in non-randomized biomedical trials and identified linguistic spin in 77% of the included studies (6) and in 26% of the abstracts of non-randomized studies (4). To date, there are no clear definitions available for assessing linguistic spin and used definitions differ between studies, which makes it difficult to meaningfully compare our findings to previously published studies on linguistic spin in biomedical literature.

Spin is one of the identified issues in biomedical research that reduces the reliability of biomedical literature. Besides spin, other problems in biomedical research affect the reliability as well. Firstly, previous research demonstrated that studies with positive or statistically significant results are more likely to be published than studies with negative or non-significant results, which is known as publication bias (32, 33). In addition, press releases are more likely to put emphasis on the beneficial effect of the investigated treatment which is associated with spin in the abstract conclusion of the article (18). Furthermore, selective reporting of favorable study outcomes, incompleteness of outcome reporting and inconsistencies in primary outcome measures between protocols and reports is a common phenomenon in trial reports (34). This may overestimate the real treatment effect that has been investigated. In particular, this is of major relevance in abstracts, because readers often seem to rely on the abstract conclusion—which is freely and easily accessible—to assess a study trial (35, 36). Moreover, Boutron et al. (7) showed that abstracts containing spin were more often rated as beneficial in contrast to abstracts without spin. Altogether, health care professionals, patients and policymakers should be aware of the possibility of overinterpretation caused by the aforementioned problems in conducting biomedical research. Furthermore, health care professionals should be trained in the proper interpretation of study results. Otherwise, it may potentially have a harmful impact on EBM in health care. Therefore, the high prevalence and extent of linguistic spin found in the present study, but also in other fields of biomedical science, has important implications for clinical practice.

The findings of the current study should be interpreted in the light of its strengths and limitations. A first strength of our study is that we have conducted a detailed and comprehensive study into linguistic spin in ophthalmologic literature based on predefined criteria. Second, to guarantee transparency of data, we presented a detailed overview of the examples we considered as linguistic spin. Third, no selection bias was introduced by including both RCTs with non-significant and significant primary outcomes. Last, because of the lack of evidence in literature on linguistic spin in RCTs about AMD, this study may provide more awareness among ophthalmologists of the possible consequences of linguistic spin on the interpretation of study outcomes. A first limitation of our study is that the interpretation of linguistic spin is subjected to individual judgements, despite the predefined criteria for the type and extent of linguistic spin. To minimize this partial subjectivity in the interpretation of linguistic spin, two authors independently completed the data extraction. Second, we defined linguistic spin as the (un)conscious use of language to convince readers of the beneficial effect of the intervention and or to suppress negative results, which is in line with the definition of spin stated in previous literature (28–30). However, there may be examples of studies in which linguistic spin has been used to emphasize the disadvantageous effect of the treatment investigated. In addition, it is important to note that we might have overestimated or underestimated the extent of linguistic spin based on our definitions used for assessing linguistic spin. For instance, we argued that not reporting *P*-values or 95% CIs for results showing a beneficial effect of the treatment investigated while claiming significance and inconsistency in the significance of the same study results between different sections of the article were examples of linguistic spin. We are aware that this is both a risk of spin, but might not be direct evidence of spin and could also be an unintended error when writing the article. Also, phrases like “trend toward” or “a positive trend” were judged to be evidence of linguistic spin, however, in some cases the use of such phrases could be justified. For example in studies with low sample sizes, because these studies are less likely to discover a statistically significant relationship due to the fact that *P*-values are affected by sample size (37, 38). Third, this study was a first attempt to provide a standardized classification system for linguistic spin. However, this way of classifying linguistic spin should be internationally scrutinized and validated.

For future research, it is relevant to determine the impact of linguistic spin on the interpretation of study results by readers and the reasons why authors (un)consciously add linguistic spin to their papers. The impact of spin on the interpretation of study results has already been investigated for spin in general by some authors (7, 17, 18), however, without reporting specifically on this subject for linguistic spin. Given the high prevalence and extent of linguistic spin in RCTs about AMD and the possible consequences for clinical decision making, efforts should be made to improve objective reporting of outcomes. This situation can be improved by trial registration in advance and providing open access to study protocols as well as full-texts of research articles (10, 34, 39, 40). Besides, researchers and clinicians should be educated on different types of spin and how spin may distort the readers' interpretation of study results (7, 40). Moreover, further research is needed to investigate linguistic spin in other study designs and other fields of research to optimize the generalizability of our findings. Last, for future researchers it could be helpful to obtain a database of linguistic spin examples together with a standardized way of classifying linguistic spin. In this way, there will be less subjectivity in assessing linguistic spin and less heterogeneity between study findings of linguistic spin. This study was a first attempt to provide such an overview and classification scheme for linguistic spin.

In conclusion, the results of this study show a high prevalence and extent of linguistic spin in abstracts (63.5%) and main texts (93.8%) of RCTs about interventions in patients diagnosed with AMD. Due to the observed prevalence and extent of linguistic spin in our study sample, we highlighted the importance of objective reporting and awareness of the possible overinterpretation caused by linguistic spin among ophthalmologists and other readers. In the future, the proposed classification scheme for linguistic spin should be validated as well as research should be conducted on whether linguistic spin distorts the reader's interpretation of study results and how this might affect EBM.

## Data availability statement

The original contributions presented in the study are included in the article/Supplementary material, further inquiries can be directed to the corresponding author.

## Author contributions

Conceptualization and design: NV, MN, LI, SI, and IS. Collection and assembly of data: NV and LI. Data analysis and interpretation: NV, LI, and FL-K. Manuscript writing: NV, MN, and IS. Critical revision of the manuscript: LI, FL-K, and SI. All authors contributed to the article and approved the submitted version.

## Conflict of interest

The authors declare that the research was conducted in the absence of any commercial or financial relationships that could be construed as a potential conflict of interest.

## Publisher's note

All claims expressed in this article are solely those of the authors and do not necessarily represent those of their affiliated organizations, or those of the publisher, the editors and the reviewers. Any product that may be evaluated in this article, or claim that may be made by its manufacturer, is not guaranteed or endorsed by the publisher.

## Abbreviations

AMD: age-related macular degeneration
CI: confidence interval
EBM: evidence-based medicine
NA: not applicable
RCT: randomized controlled trial
ref: reference group
sign.: significant or significance.
