# Supplementary material for: Linguistic spin in randomized controlled trials about age-related macular degeneration
Source: Front Epidemiol. 2022 Oct 31;2:961996. doi: 10.3389/fepid.2022.961996 (PMC10910936; doi:10.3389/fepid.2022.961996)
Supplement: Supplementary file 1 [file Table_1.DOCX]

**Supplementary A. Search strategy for the PubMed database**

(Macular degeneration[MeSH Terms]) OR Macular degeneration*[Title/Abstract]) OR Degeneration*, macular[Title/Abstract]) OR Maculopath*[Title/Abstract]) OR Macular dystroph*[Title/Abstract]) OR Dystroph*, macular[Title/Abstract]) OR AMD[Title/Abstract]) OR ARMD[Title/Abstract].
