# Supplementary material for: Linguistic spin in randomized controlled trials about age-related macular degeneration
Source: Front Epidemiol. 2022 Oct 31;2:961996. doi: 10.3389/fepid.2022.961996 (PMC10910936; doi:10.3389/fepid.2022.961996)
Supplement: Supplementary file 2 [file Table_2.DOCX]

**Supplementary Table B. Overview of linguistic spin categories together with examples provided from the included RCTs.**

| Linguistic spin category | | Examples/description |
| --- | --- | --- |
| 1 | Use of words to reject or explain non-statistically significant results | ‘Nonsignificant tendency’, ‘tendency toward improvement’, ‘near improvement’ |
| 2 | Use of words to claim comparable effectiveness or equivalence despite a *P*-value above 0.05 | ‘Statistically borderline difference’, ‘statistically similar’ |
| 3 | Use of words to point out the beneficial effect of the treatment investigated (e.g. ‘a positive trend’ or ‘excellent’ results) | ‘Tendency toward’, ‘very impressive’, ‘highly significant’ |
| 4 | Use of ‘(statistically) significant/significance’ without reporting a *P*-value or a 95% CI for results showing a beneficial effect of the treatment investigated | ‘Statistically significant’, ‘significant reduction’, ‘significant increase’ |
| 5 | Particular focus on results with statistical significance in abstract and or main text (i.e. only pointing out the statistically significant results) | A secondary outcome is discussed in the abstract instead of the primary outcome because only the secondary outcome is statistically significant. |
| 6 | Inconsistency in the significance reported for the same results within the article | A different *P*-value is reported for the same outcome variable in another section of the article. E.g. for BCVA improvement in study group 1 a *P*-value of 0.03 is reported in the abstract and a *P*-value of 0.047 is reported in the results section of the main text. |
| 7 | Other forms of linguistic spin | ‘This is the first study’, ‘has the potential to influence’, ‘these findings are important’ |

*NA: not applicable.*
