# Supplementary material for: Linguistic spin in randomized controlled trials about age-related macular degeneration
Source: Front Epidemiol. 2022 Oct 31;2:961996. doi: 10.3389/fepid.2022.961996 (PMC10910936; doi:10.3389/fepid.2022.961996)
Supplement: Supplementary file 3 [file Table_3.DOCX]

**Supplementary Table C1. Baseline characteristics of included RCTs about age-related macular degeneration**

|  | Author | Country | Journal | Endorsing CONSORT | Impact factor | Intervention | Comparator | Number of treatment arms | Sample size (patients/eyes) |
| --- | --- | --- | --- | --- | --- | --- | --- | --- | --- |
| 1 | Ahmadieh, 2011 (1) | Iran | Retina | No | 2,812 | Combined therapy | Drug | 2 | 120/120 |
| 2 | Brody, 2011 (2) | United States of America | Annals of Clinical Psychiatry | No | 1,490 | Drug | Placebo | 2 | 18/18 |
| 3 | Blaha, 2011 (3) | United States of America | Retina | No | 2,812 | Drug | Drug | 4 | 24/NR |
| 4 | Dunavoelgyi, 2011 (4) | Austria | Acta Ophthalmologica | No | 2,629 | Combined therapy | Phototherapy | 2 | 40/40 |
| 5 | Giustolisi, 2011 (5) | Italy | Digital Journal of Ophthalmology | No | Not available | Combined therapy | Drug | 2 | 47/47 |
| 6 | Falkner/Radler, 2011 (6) | Austria | British Journal of Ophthalmology | Yes | 2,902 | Surgery | Surgery | 2 | 14/NR |
| 7 | Holton, 2011 (7) | Denmark | Acta Ophthalmologica | No | 2,629 | Other | Other | 2 | 70/70 |
| 8 | Seiple, 2011 (8) | United States of America | Investigative Ophthalmology and Visual Science | No | 3,597 | Other | Placebo | 3 | 30/30 |
| 9 | Weigert, 2011 (9) | Austria | Investigative Ophthalmology and Visual Science | No | 3,597 | Supplement | Placebo | 2 | 126/126 |
| 10 | Richer, 2011 (10) | United States of America | Optometry | No | 0,741 | Supplement | Supplement | 3 | 60/119 |
| 11 | Rencova, 2011 (11) | Czech Republic | Acta Ophthalmologica | No | 2,629 | Other | Usual care | 2 | 32/54 |
| 12 | Schmid/Kubista, 2011 (12) | Austria | Current Eye Research | No | 1,280 | Drug | Combination therapy | 3 | 54/54 |
| 13 | Borelli, 2012 (13) | Italy | International Journal of Ophthalmology | No | 0,119 | Other | Usual care | 2 | 140/140 |
| 14 | Cohen, 2012 (14) | Spain, Belgium, France | Retina | No | 2,825 | Drug | Placebo | 2 | 1192/1192 |
| 15 | El-Mollayess, 2012 (15) | Lebanon | American Journal of Ophthalmology | Yes | 3,631 | Care mangement | Care management | 2 | 60/120 |
| 16 | Georgakopoulos, 2012 (16) | Greece | Journal of Ocular Pharmacology and Therapeutics | No | 1,293 | Drug | Placebo | 2 | 30/30 |
| 17 | Gomi, 2012 (17) | Japan | Retina | No | 2,825 | Combined therapy | Placebo | 2 | 38/38 |
| 18 | Li, 2012 (18) | China | Ophthalmology | Yes | 5,563 | Care mangement | Care management | 2 | 185/185 |
| 19 | Ma, 2012 (19) | China | American Journal of Ophthalmology | Yes | 3,631 | Supplement | Placebo | 4 | 108/108 |
| 20 | Ma, 2012 (20) | China | Opthalmology | Yes | 5,563 | Supplement | Placebo | 4 | 108/108 |
| 21 | Ozturk, 2012 (21) | Turkey | International Journal of Ophthalmology | No | 0,119 | Combined therapy | Drug | 3 | 45/45 |
| 22 | Piermarocchi, 2012 (22) | Italy | European Journal of Ophthalmology | No | 0,912 | Supplement | Usual care | 2 | 145/145 |
| 23 | Rifkin, 2012 (23) | USA | European Journal of Ophthalmology | No | 0,912 | Drug | Usual care | 3 | 40/40 |
| 24 | Söderberg, 2012 (24) | Sweden | British Journal of Ophthalmology | Yes | 2,725 | Combined therapy | Placebo | 2 | 100/100 |
| 25 | Sun, 2012 (25) | China | Annals of Nutrition and Metabolism | No | 1,661 | Supplement | Placebo | 2 | 60/NR |
| 26 | Williams, 2012 (26) | United States of America | Clinical Ophthalmology | No | 1,660 | Combined therapy | Drug | 2 | 60/60 |
| 27 | Anastassiou, 2013 (27) | Germany | Restorative Neurology and Neuroscience | No | 4,179 | Device | Placebo | 2 | 22/22 |
| 28 | Arnold, 2013 (28) | Germany | Nutrition | No | 3,046 | Supplement | Placebo | 2 | 20/20 |
| 29 | Berrow, 2013 (29) | United Kingdom | British Journal of Nutrition | Yes | 3,342 | Supplement | Usual care | 2 | 16/16 |
| 30 | Blaha, 2013 (30) | Czech Republic | Atherosclerosis Supplements | No | 9,667 | Other | Usual care | 2 | 72/NR |
| 31 | Dawczynski, 2013 (31) | Germany | Graefe's Archive for Clinical and Experimental Ophthalmology | No | 2,333 | Supplement | Placebo | 3 | 172/127 |
| 32 | García-Layana, 2013 (32) | Spain | Nutrients | No | 3,148 | Supplement | Placebo | 2 | 44/NR |
| 33 | Guymer, 2013 (33) | Australia | PLoS One | Yes | 3,534 | Drug | Placebo | 2 | 114/179 |
| 34 | Huang, 2013 (34) | China | Nutrition | No | 3,046 | Supplement | Placebo | 4 | 108/108 |
| 35 | Jackson, 2013 (35) | 21 Europian sites | Ophthalmology | Yes | 6,170 | Radiotherapy | Drug | 3 | 230/230 |
| 36 | Krebs, 2013 (36) | Austria | British Journal of Ophthalmology | Yes | 2,809 | Drug | Drug | 2 | 321/321 |
| 37 | Krebs, 2013 (37) | Austria | Acta Opthalmologica | No | 2,512 | Combined therapy | Drug | 2 | 51/51 |
| 38 | Menon, 2013 (38) | United Kingdom | Eye | Yes | 1,897 | Care mangement | Usual care | 2 | 100/100 |
| 39 | Murray, 2013 (39) | United Kingdom, The Netherlands | Investigative Ophthalmology and Visual Science | No | 3,661 | Supplement | Placebo | 2 | 84/84 |
| 40 | Rovner, 2013 (40) | United States | Ophthalmology | Yes | 6,170 | Care mangement | Care management | 2 | 241/241 |
| 41 | Russo, 2013 (41) | Italy | British Journal of Ophthalmology | Yes | 2,809 | Combined therapy | Drug | 2 | 56/56 |
| 42 | Souied, 2013 (42) | France | Ophthalmology | Yes | 6,170 | Supplement | Placebo | 2 | 300/300 |
| 43 | Vingolo, 2013 (43) | Italy | Applied Psychophysiology and Biofeedback | No | 1,593 | Other | Other | 2 | 30/30 |
| 44 | Chew, 2014 (44) | United States | Ophthalmology | Yes | 6,135 | Care mangement | Usual care | 2 | 1520/2586 |
| 45 | Bittner, 2014 (45) | United States | Journal of Clinical & Experimental Ophthalmology | No | 1,420 | Care mangement | Usual care | 2 | 198/198 |
| 46 | Piri, 2014 (46) | Iran | Journal of Ophthalmic and Vision Research | No | 0,940 | Combined therapy | Combination therapy | 2 | 84/84 |
| 47 | Rezende, 2014 (47) | Canada | American Journal of Ophthalmology | Yes | 3,871 | Supplement | Supplement | 3 | 30/NR |
| 48 | Rovner, 2014 (48) | United States of America | Ophthalmology | Yes | 6,135 | Care mangement | Care management | 2 | 188/188 |
| 49 | Sabour-Pickett, 2014 (49) | Ireland | Retina | No | 3,243 | Supplement | Supplement | 3 | 67/67 |
| 50 | Scholler, 2014 (50) | Austria | Wiener Klinische Wochenschrift | No | 0,836 | Drug | Drug | 2 | 55/55 |
| 51 | Schramm, 2014 (51) | Germany | Acta Ophthalmologica | No | 2,844 | Combined therapy | Drug | 2 | 50/50 |
| 52 | Selim, 2014 (52) | Turkey | Turkish Journal of Medical Sciences | No | 0,497 | Combined therapy | Phototherapy | 2 | 80/80 |
| 53 | Sin, 2014 (53) | Czech Republic | Biomedical Papers | No | 1,200 | Drug | Placebo | 2 | 60/60 |
| 54 | Sizmaz, 2014 (54) | Turkey | European Journal of Ophthalmology | No | 1,068 | Drug | Drug | 2 | 40/40 |
| 55 | Barikian, 2015 (55) | Lebanon | American Journal of Ophthalmology | Yes | 3,831 | Care mangement | Care management | 3 | 90/90 |
| 56 | Huang, 2015 (56) | China | British Journal of Ophthalmology | Yes | 3,036 | Supplement | Placebo | 4 | 112/NR |
| 57 | Datseris, 2015 (57) | Greece | Seminars in Ophthalmology | No | 1,184 | Combined therapy | Drug | 2 | 100/100 |
| 58 | Hatz, 2015 (58) | Switzerland | Ophthalmologica | No | 1,515 | Combined therapy | Usual care | 2 | 40/NR |
| 59 | Kuppermann, 2015 (59) | Australia, France, Israel, Italy, Korea, New Zealand, Portugal, United Kingdom, United States | Ophthalmologica | No | 1,515 | Drug | Placebo | 2 | 243/243 |
| 60 | Li, 2015 (60) | Canada | JAMA ophthalmology | No | 4,340 | Care mangement | Usual care | 2 | 169/19 |
| 61 | Markun, 2015 (61) | Switzerland | PLoS ONE | Yes | 3,057 | Care mangement | Usual care | 2 | 169/190 |
| 62 | Semeraro, 2015 (62) | Italy | Retina | No | 3,039 | Combined therapy | Drug | 3 | 75/75 |
| 63 | Wolf Schnurrbusch, 2015 (63) | Switzerland | Investigative Ophthalmology and Visual Science | No | 3,427 | Supplement | Supplement | 2 | 79/79 |
| 64 | Zehetner, 2015 (64) | Austria | Acta Ophthalmologica | No | 3,032 | Drug | Drug | 2 | 38/38 |
| 65 | Lashay, 2016 (65) | Iran | Medical hypothesis, discovery & innovation ophthalmology journal | No | 0,960 | Supplement | Placebo | 2 | 60/60 |
| 66 | Rezar-Dreindl, 2016 (66) | Austria | Investigative Ophthalmology & Visual Science | No | 3,303 | Combined therapy | Drug | 2 | 40/40 |
| 67 | Saviano, 2016 (67) | Italy | Digital Journal of Ophthalmology | No | 0,130 | Combined therapy | Drug | 2 | 62/62 |
| 68 | Tao, 2016 (68) | China | Tohoku Journal of Experimental Medicine | No | 1,278 | Supplement | Placebo | 2 | 100/NR |
| 69 | Weingessel, 2016 (69) | Austria | Wiener klinische Wochenschrift | No | 0,974 | Combined therapy | Drug | 2 | 34/34 |
| 70 | Dong, 2016 (70) | China | Experimental and Therapeutic Medicine | No | 1,261 | Combined therapy | Drug | 2 | 96/192 |
| 71 | Mantel, 2016 (71) | Switzerland | Retina | No | 3,700 | Care mangement | Drug | 2 | 19/21 |
| 72 | Abdelfattah, 2017 (72) | United States of America | Ophthalmology | Yes | 7,479 | Care mangement | Care management | 2 | 60/60 |
| 73 | Akuffo, 2017 (73) | Ireland | Investigative ophthalmology & visual science | No | 3,388 | Supplement | Supplement | 2 | 121/121 |
| 74 | Mori, 2017 (74) | Japan | Ophthalmologica | No | 1,605 | Care mangement | Usual care | 2 | 70/70 |
| 75 | Sengul, 2017 (75) | Turkey | Eye | Yes | 2,478 | Drug | Drug | 2 | 72/72 |
| 76 | Azar, 2017 (76) | France, Lebanon | Journal Francais d'Ophtalmologie | No | 0,460 | Supplement | Placebo | 2 | 126/NR |
| 77 | Figurska, 2018 (77) | Poland | Medical Science Monitor | No | 1,980 | Surgery | Usual care | 2 | 49/50 |
| 78 | Li, 2018 (78) | China | International Journal of Ophthalmology | No | 1,189 | Supplement | Usual care | 2 | 114/114 |
| 79 | Motarjemizadeh, 2018 (79) | Iran | Middle East African Journal of Ophthalmology | No | 1,080 | Combined therapy | Drug | 2 | 142/142 |
| 80 | Rosenfeld, 2018 (80) | Germany, USA | Ophthalmology | Yes | 7,732 | Drug | Placebo | 4 | 508/508 |
| 81 | Russo, 2018 (81) | Italy | Clinical Ophthalmology | No | 2,040 | Combined therapy | Drug | 2 | 58/58 |
| 82 | Giancipoli, 2018 (82) | Italy | Journal of Ophthalmology | No | 1,580 | Combined therapy | Drug | 2 | 15/16 |
| 83 | Broadhead, 2018 (83) | Australia | Graefe's Archive for Clinical and Experimental Ophthalmology | No | 2,396 | Combined therapy | Placebo | 2 | 100/167 |
| 84 | Guymer, 2019 (84) | Australia, Nothern Ireland | Ophthalmology | Yes | 8,470 | Radiotherapy | Placebo | 2 | 292/584 |
| 85 | Kaltenegger, 2019 (85) | Germany | Graefe's archive for clinical and experimental ophthalmology | No | 2,396 | Care mangement | Placebo | 2 | 52/52 |
| 86 | Liu, 2019 (86) | China | American Journal of Ophthalmology | Yes | 4,013 | Drug | Placebo | 2 | 124/124 |
| 87 | Luo, 2019 (87) | China | Journal of Traditional Chinese Medicine | No | 0,676 | Combined therapy | Drug | 2 | 75/75 |
| 88 | Markowitz, 2019 (88) | Canada | Retina | No | 3,649 | Radiotherapy | Placebo | 2 | 30/46 |
| 89 | Nunes, 2019 (89) | Brazil | Arquivos Brasileiros de Oftalmologia | No | 0,617 | Care mangement | Care management | 3 | 45/45 |
| 90 | Parravano, 2019 (90) | Italy | Advances in Therapy | Yes | 3,871 | Supplement | Placebo | 2 | 30/30 |
| 91 | Semeraro, 2019 (91) | Italy | British Journal of Clinical Pharmacology | No | 3,740 | Combined therapy | Drug | 3 | 60/60 |
| 92 | Yuan, 2019 (92) | China | Experimental and Therapeutic Medicine | No | 1,785 | Drug | Drug | 2 | 80/80 |
| 93 | Gillies, 2020 (93) | Australia | Ophthalmology | Yes | 7,732 | Drug | Drug | 2 | 281/281 |
| 94 | Hsu, 2020 (94) | United States of America | JAMA Ophthalmology | No | 6,198 | Drug | Placebo | 2 | 52/52 |
| 95 | Piatti, 2020 (95) | Italy | European Journal of Ophthalmology | No | 1,642 | Supplement | Placebo | 2 | 80/80 |
| 96 | Sawa, 2020 (96) | Japan | Scientific Reports | No | 3,998 | Supplement | Supplement | 2 | 42/42 |

*Abbreviations: NR: not reported.*

**Supplementary Table C2. Baseline characteristics of included RCTs about age-related macular degeneration.**

|  | Author | Funding | Drop-out (%) | International collaboration | Number of citations | Type primary outcome | Study group* |
| --- | --- | --- | --- | --- | --- | --- | --- |
| 1 | Ahmadieh, 2011 (1) | Non-profit | < 20 | No | 17 | Efficacy | 2 |
| 2 | Brody, 2011 (2) | For profit | < 20 | No | 7 | Efficacy | 1 |
| 3 | Blaha, 2011 (3) | NR | < 20 | No | 45 | Efficacy | 2 |
| 4 | Dunavoelgyi, 2011 (4) | For profit | < 20 | No | 6 | NA | 3 |
| 5 | Giustolisi, 2011 (5) | Non-profit | < 20 | No | NR | NA | 3 |
| 6 | Falkner/Radler, 2011 (6) | NR | < 20 | No | 88 | NA | 3 |
| 7 | Holton, 2011 (7) | For profit | < 20 | No | 3 | Efficacy | 1 |
| 8 | Seiple, 2011 (8) | Non-profit | < 20 | No | 48 | Efficacy | 1 |
| 9 | Weigert, 2011 (9) | For profit | 20-30 | No | 87 | Efficacy | 1 |
| 10 | Richer, 2011 (10) | For profit | < 20 | No | 57 | Efficacy | 2 |
| 11 | Rencova, 2011 (11) | Non-profit | < 20 | No | 11 | NA | 3 |
| 12 | Schmid/Kubista, 2011 (12) | NR | < 20 | No | 7 | NA | 3 |
| 13 | Borelli, 2012 (13) | NR | < 20 | No | 20 | Efficacy | 2 |
| 14 | Cohen, 2012 (14) | For profit | 20-30 | Yes | 9 | Efficacy | 2 |
| 15 | El-Mollayess, 2012 (15) | Non-profit | < 20 | No | 17 | NA | 3 |
| 16 | Georgakopoulos, 2012 (16) | NR | < 20 | No | 8 | Efficacy | 1 |
| 17 | Gomi, 2012 (17) | Non-profit | < 20 | No | 28 | Efficacy | 1 |
| 18 | Li, 2012 (18) | Non-profit | < 20 | No | 18 | Efficacy | 2 |
| 19 | Ma, 2012 (19) | Non-profit | < 20 | No | 52 | NA | 3 |
| 20 | Ma, 2012 (20) | Non-profit | < 20 | No | 96 | Efficacy | 2 |
| 21 | Ozturk, 2012 (21) | NR | < 20 | No | 2 | NA | 3 |
| 22 | Piermarocchi, 2012 (22) | Non-profit | 20-30 | No | 50 | Safety | 1 |
| 23 | Rifkin, 2012 (23) | Non-profit | < 20 | No | 11 | Efficacy | 1 |
| 24 | Söderberg, 2012 (24) | NR | 20-30 | No | 13 | Efficacy | 1 |
| 25 | Sun, 2012 (25) | Non-profit/for profit | < 20 | No | 16 | NA | 3 |
| 26 | Williams, 2012 (26) | For profit | < 20 | No | 11 | NA | 3 |
| 27 | Anastassiou, 2013 (27) | NR | < 20 | No | 18 | Efficacy | 2 |
| 28 | Arnold, 2013 (28) | Non-profit | < 20 | No | 10 | NA | 3 |
| 29 | Berrow, 2013 (29) | For profit | < 20 | No | 15 | Efficacy | 2 |
| 30 | Blaha, 2013 (30) | Non-profit | < 20 | No | 12 | NA | 3 |
| 31 | Dawczynski, 2013 (31) | For profit | < 20 | No | 51 | NA | 3 |
| 32 | García-Layana, 2013 (32) | Non-profit | < 20 | No | 20 | Efficacy | 1 |
| 33 | Guymer, 2013 (33) | Non-profit/for profit | < 20 | No | 31 | Efficacy | 2 |
| 34 | Huang, 2013 (34) | Non-profit | < 20 | No | 19 | NA | 3 |
| 35 | Jackson, 2013 (35) | For profit | < 20 | Yes | 38 | Efficacy | 1 |
| 36 | Krebs, 2013 (36) | Unclear | < 20 | No | 122 | Efficacy | 2 |
| 37 | Krebs, 2013 (37) | For profit | < 20 | No | 18 | Efficacy | 2 |
| 38 | Menon, 2013 (38) | NR | < 20 | No | 14 | NA | 3 |
| 39 | Murray, 2013 (39) | Non-profit | < 20 | Yes | 53 | NA | 3 |
| 40 | Rovner, 2013 (40) | Non-profit | < 20 | No | 24 | Efficacy | 2 |
| 41 | Russo, 2013 (41) | NR | < 20 | No | 12 | Efficacy | 2 |
| 42 | Souied, 2013 (42) | For profit | 20-30 | No | 63 | Efficacy | 2 |
| 43 | Vingolo, 2013 (43) | Non-profit | < 20 | No | 12 | NA | 3 |
| 44 | Chew, 2014 (44) | Unclear | < 20 | No | 72 | Efficacy | 1 |
| 45 | Bittner, 2014 (45) | Non-profit | > 30 | No | NR | NA | 3 |
| 46 | Piri, 2014 (46) | Non-profit | < 20 | No | 3 | Efficacy | 2 |
| 47 | Rezende, 2014 (47) | Non-profit/for profit | 20-30 | No | 18 | Efficacy | 1 |
| 48 | Rovner, 2014 (48) | Non-profit | < 20 | No | 59 | Efficacy | 2 |
| 49 | Sabour-Pickett, 2014 (49) | Non-profit | 20-30 | No | 30 | NA | 3 |
| 50 | Scholler, 2014 (50) | NR | < 20 | No | 3 | Efficacy | 2 |
| 51 | Schramm, 2014 (51) | Non-profit | < 20 | No | 5 | Efficacy | 2 |
| 52 | Selim, 2014 (52) | NR | < 20 | No | 3 | Efficacy | 2 |
| 53 | Sin, 2014 (53) | Non-profit | < 20 | No | 3 | Efficacy | 2 |
| 54 | Sizmaz, 2014 (54) | Non-profit | < 20 | No | 17 | NA | 3 |
| 55 | Barikian, 2015 (55) | Non-profit | < 20 | No | 11 | Efficacy | 1 |
| 56 | Huang, 2015 (56) | Non-profit | < 20 | No | 17 | Efficacy | 2 |
| 57 | Datseris, 2015 (57) | NR | < 20 | No | 7 | 4 | 3 |
| 58 | Hatz, 2015 (58) | For profit | < 20 | No | 13 | NA | 3 |
| 59 | Kuppermann, 2015 (59) | For profit | < 20 | No | 15 | Efficacy | 1 |
| 60 | Li, 2015 (60) | Non-profit | < 20 | No | 21 | NA | 3 |
| 61 | Markun, 2015 (61) | Non-profit | < 20 | No | 4 | Efficacy | 2 |
| 62 | Semeraro, 2015 (62) | NR | < 20 | No | 14 | NA | 3 |
| 63 | Wolf Schnurrbusch, 2015 (63) | Non-profit/for profit | < 20 | No | 16 | NA | 3 |
| 64 | Zehetner, 2015 (64) | NR | < 20 | No | 46 | Efficacy | 1 |
| 65 | Lashay, 2016 (65) | Non-profit | > 30 | No | NR | NA | 3 |
| 66 | Rezar-Dreindl, 2016 (66) | Non-profit | < 20 | No | 28 | NA | 3 |
| 67 | Saviano, 2016 (67) | NR | < 20 | No | 6 | Efficacy | 1 |
| 68 | Tao, 2016 (68) | Non-profit | < 20 | No | 6 | NA | 3 |
| 69 | Weingessel, 2016 (69) | NR | < 20 | No | 2 | NA | 3 |
| 70 | Dong, 2016 (70) | NR | < 20 | No | 5 | NA | 3 |
| 71 | Mantel, 2016 (71) | NR | < 20 | No | 17 | NA | 3 |
| 72 | Abdelfattah, 2017 (72) | Non-profit | 20-30 | No | 33 | Efficacy | 2 |
| 73 | Akuffo, 2017 (73) | Non-profit | < 20 | No | 10 | Efficacy | 2 |
| 74 | Mori, 2017 (74) | Non-profit | < 20 | No | 4 | NA | 3 |
| 75 | Sengul, 2017 (75) | NR | < 20 | No | 4 | NA | 3 |
| 76 | Azar, 2017 (76) | Non-profit | < 20 | No | 5 | Efficacy | 2 |
| 77 | Figurska, 2018 (77) | Non-profit | < 20 | No | 0 | NA | 3 |
| 78 | Li, 2018 (78) | Non-profit | < 20 | No | 5 | NA | 3 |
| 79 | Motarjemizadeh, 2018 (79) | Non-profit | < 20 | No | 1 | NA | 3 |
| 80 | Rosenfeld, 2018 (80) | For profit | > 30 | Yes | 24 | Efficacy | 2 |
| 81 | Russo, 2018 (81) | Non-profit | < 20 | No | 0 | NA | 3 |
| 82 | Giancipoli, 2018 (82) | For profit | < 20 | No | 2 | Efficacy | 1 |
| 83 | Broadhead, 2018 (83) | Non-profit | < 20 | No | 6 | Efficacy | 1 |
| 84 | Guymer, 2019 (84) | Non-profit/for profit | < 20 | Yes | 20 | Efficacy | 2 |
| 85 | Kaltenegger, 2019 (85) | Non-profit | 20-30 | No | 1 | Efficacy | 4 |
| 86 | Liu, 2019 (86) | For profit | < 20 | No | 23 | Efficacy | 1 |
| 87 | Luo, 2019 (87) | Non-profit | < 20 | No | 0 | NA | 3 |
| 88 | Markowitz, 2019 (88) | Non-profit | < 20 | No | 1 | NA | 3 |
| 89 | Nunes, 2019 (89) | Non-profit | < 20 | No | 1 | NA | 3 |
| 90 | Parravano, 2019 (90) | Non-profit/for profit | < 20 | No | 0 | Efficacy | 4 |
| 91 | Semeraro, 2019 (91) | Non-profit | < 20 | No | 1 | NA | 3 |
| 92 | Yuan, 2019 (92) | Non-profit | < 20 | No | NR | NA | 3 |
| 93 | Gillies, 2020 (93) | For profit | < 20 | No | 1 | Efficacy | 2 |
| 94 | Hsu, 2020 (94) | Non-profit | < 20 | No | 1 | Efficacy | 1 |
| 95 | Piatti, 2020 (95) | Non-profit | < 20 | No | 0 | Efficacy | 1 |
| 96 | Sawa, 2020 (96) | Non-profit/for profit | < 20 | No | 0 | Efficacy | 2 |

*Abbreviations: NA: not applicable; NR: not reported.*

** Study group 1: studies with a statistically significant result for the primary outcome. Study group 2: studies with a statistically non-significant result for the primary outcome. Study group 3: studies in which a primary outcome was not clearly specified. Study group 4: studies in which the significance of the primary outcome was not reported.*

**References**

1. Ahmadieh H, Taei R, Riazi-Esfahani M, Piri N, Homayouni M, Daftarian N, et al. Intravitreal bevacizumab versus combined intravitreal bevacizumab and triamcinolone for neovascular age-related macular degeneration: Six-month results of a randomized clinical trial. Retina. 2011;31(9):1819–26.

2. Brody B. Treatment of Depression Associated with Age-related Macular Degeneration in a Double-Blind Randomized Controlled Study. Physiol Behav. 2011;176(1):139–48.

3. Blaha GR, Tilton EP, Barouch FC, Marx JL. Randomized trial of anesthetic methods for intravitreal injections. Retina. 2011;31(3):535–9.

4. Dunavoelgyi R, Sacu S, Simader C, Pruente C, Schmidt-Erfurth U. Changes in macular sensitivity after reduced fluence photodynamic therapy combined with intravitreal triamcinolone. Acta Ophthalmol. 2011;89(2):166–71.

5. Giustolisi. Combined intravitreal ranibizumab and verteporfin photodynamic therapy versus ranibizumab alone for the treatment of age-related macular degeneration. Digit J Ophthalmol. 2011;

6. Falkner-Radler CI, Krebs I, Glittenberg C, Považay B, Drexler W, Graf A, et al. Human retinal pigment epithelium (RPE) transplantation: Outcome after autologous RPE-choroid sheet and RPE cell-suspension in a randomised clinical study. Br J Ophthalmol. 2011;95(3):370–5.

7. Holton H, Christiansen AB, Albeck MJ, Johnsen CR. The impact of light source on discrimination ability in subjects with age-related macular degeneration. Acta Ophthalmol. 2011;89(8):779–84.

8. Seiple W, Grant P, Szlyk JP. Reading rehabilitation of individuals with AMD: Relative effectiveness of training approaches. Investig Ophthalmol Vis Sci. 2011;52(6):2938–44.

9. Weigert G, Kaya S, Pemp B, Sacu S, Lasta M, Werkmeister RM, et al. Effects of lutein supplementation on macular pigment optical density and visual acuity in patients with age-related macular degeneration. Investig Ophthalmol Vis Sci. 2011;52(11):8174–8.

10. Richer SP, Stiles W, Graham-Hoffman K, Levin M, Ruskin D, Wrobel J, et al. Randomized, double-blind, placebo-controlled study of zeaxanthin and visual function in patients with atrophic age-related macular degeneration: The Zeaxanthin and Visual Function Study (ZVF) FDA IND #78, 973. Optometry [Internet]. 2011;82(11):667-680.e6. Available from: http://dx.doi.org/10.1016/j.optm.2011.08.008

11. Rencová E, Bláha M, Studnička J, Blažek M, Bláha V, Dusová J, et al. Haemorheopheresis could block the progression of the dry form of age-related macular degeneration with soft drusen to the neovascular form. Acta Ophthalmol. 2011;89(5):463–71.

12. Schmid-Kubista KE, Krebs I, Ansari-Shahrezaei S, Haas P, Hagen S, Binder S. Comparing treatment of neovascular age-related macular degeneration with sequential intravitreal avastin and macugen versus intravitreal mono-therapy - A pilot study. Curr Eye Res. 2011;36(10):958–63.

13. Borrelli E, Diadori A, Zalaffi A, Bocci V. Effects of major ozonated autohemotherapy in the treatment of dry age related macular degeneration: a randomized controlled clinical study. Int J Ophthalmol [Internet]. 2012;5(6):708–13. Available from: https://www.ncbi.nlm.nih.gov/pubmed/23275905

14. Cohen S-Y, Bourgeois H, Corbe C, Chaine G, Espinasse-Berrod M-A, Garcia-Sanchez J, et al. Randomized clinical trial France DMLA2: effect of trimetazidine on exudative and nonexudative age-relatedmacular degeneration. Retina [Internet]. 2012;32(4):834–43. Available from: https://www.ncbi.nlm.nih.gov/pubmed/21822162

15. El-Mollayess GM, Mahfoud Z, Schakal AR, Salti HI, Jaafar D, Bashshur ZF. Fixed-interval versus OCT-guided variable dosing of intravitreal bevacizumab in the management of neovascular age-related macular degeneration: a 12-month randomized prospective study. Am J Ophthalmol [Internet]. 2012;153(3):481-489.e1. Available from: https://www.ncbi.nlm.nih.gov/pubmed/22014603

16. Georgakopoulos CD, Vasilakis PT, Makri OE, Beredima E, Pharmakakis NM. Effect of ketorolac 0.5% drops on patients’ pain perception during intravitreal injection procedure. J Ocul Pharmacol Ther [Internet]. 2012;28(5):455–8. Available from: https://www.ncbi.nlm.nih.gov/pubmed/22587573

17. Gomi F, Sawa M, Tsujikawa M, Nishida K. Topical bromfenac as an adjunctive treatment with intravitreal ranibizumab for exudative age-related macular degeneration. Retina. 2012;32(9):1804–10.

18. Li X, Hu Y, Sun X, Zhang J, Zhang M, Neovascular Age-Related Macular Degeneration Treatment Trial Using Bevacizumab (NATTB) [Collective Name]. Bevacizumab for neovascular age-related macular degeneration in China. Ophthalmology [Internet]. 2012;119(10):2087–93. Available from: https://www.ncbi.nlm.nih.gov/pubmed/22818896

19. Ma L, Dou H-L, Huang Y-M, Lu X-R, Xu X-R, Qian F, et al. Improvement of retinal function in early age-related macular degeneration after lutein and zeaxanthin supplementation: a randomized, double-masked, placebo-controlled trial. Am J Ophthalmol [Internet]. 2012;154(4):625-634.e1. Available from: https://www.ncbi.nlm.nih.gov/pubmed/22835510

20. Ma L, Yan S-F, Huang Y-M, Lu X-R, Qian F, Pang H-L, et al. Effect of lutein and zeaxanthin on macular pigment and visual function in patients with early age-related macular degeneration. Ophthalmology [Internet]. 2012;119(11):2290–7. Available from: https://www.ncbi.nlm.nih.gov/pubmed/22858124

21. Ozturk T, Oner H, Saatci AO, Kaynak S. Low-fluence photodynamic therapy combinations in the treatment of exudative age-related macular degeneration. Int J Ophthalmol. 2012;5(3):377–83.

22. Piermarocchi S, Saviano S, Parisi V, Tedeschi M, Panozzo G, Scarpa G, et al. Carotenoids in Age-related Maculopathy Italian Study (CARMIS): two-year results of a randomized study. Eur J Ophthalmol [Internet]. 2012;22(2):216–25. Available from: https://www.ncbi.nlm.nih.gov/pubmed/22009916

23. Rifkin L, Schaal S. Shortening ocular pain duration following intravitreal injections. Eur J Ophthalmol [Internet]. 2012;22(6):1008–12. Available from: https://www.ncbi.nlm.nih.gov/pubmed/22562296

24. Söderberg AC, Algvere P V., Hengstler JC, Söderberg P, Seregard S, Kvanta A. Combination therapy with low-dose transpupillary thermotherapy and intravitreal ranibizumab for neovascular age-related macular degeneration: A 24-month prospective randomised clinical study. Br J Ophthalmol. 2012;96(5):714–8.

25. Sun Y-D, Dong Y-D, Fan R, Zhai L-L, Bai Y-L, Jia L-H. Effect of (R)-α-lipoic acid supplementation on serum lipids and antioxidative ability in patients with age-related macular degeneration. Ann Nutr Metab [Internet]. 2012;60(4):293–7. Available from: https://www.ncbi.nlm.nih.gov/pubmed/22678104

26. Williams PD, Callanan D, Solley W, Avery RL, Pieramici DJ, Aaberg T. A prospective pilot study comparing combined intravitreal ranibizumab and half-fluence photodynamic therapy with ranibizumab monotherapy in the treatment of neovascular age-related macular degeneration. Clin Ophthalmol [Internet]. 2012;6:1519–25. Available from: https://www.ncbi.nlm.nih.gov/pubmed/23055673

27. Anastassiou G, Schneegans A-L, Selbach M, Kremmer S. Transpalpebral electrotherapy for dry age-related macular degeneration (AMD): an exploratory trial. Restor Neurol Neurosci [Internet]. 2013;31(5):571–8. Available from: https://www.ncbi.nlm.nih.gov/pubmed/23760223

28. Arnold C, Jentsch S, Dawczynski J, Böhm V. Age-related macular degeneration: Effects of a short-term intervention with an oleaginous kale extract--a pilot study. Nutrition [Internet]. 2013;29(11):1412–7. Available from: https://www.ncbi.nlm.nih.gov/pubmed/24103519

29. Berrow EJ, Bartlett HE, Eperjesi F, Gibson JM. The effects of a lutein-based supplement on objective and subjective measures of retinal and visual function in eyes with age-related maculopathy -- a randomised controlled trial. Br J Nutr [Internet]. 2013;109(11):2008–14. Available from: https://www.ncbi.nlm.nih.gov/pubmed/23084077

30. Blaha M, Rencova E, Langrova H, Studnicka J, Blaha V, Rozsival P, et al. Rheohaemapheresis in the treatment of nonvascular age-related macular degeneration. Atheroscler Suppl [Internet]. 2013;14(1):179–84. Available from: https://www.ncbi.nlm.nih.gov/pubmed/23357162

31. Dawczynski J, Jentsch S, Schweitzer D, Hammer M, Lang GE, Strobel J. Long term effects of lutein, zeaxanthin and omega-3-LCPUFAs supplementation on optical density of macular pigment in AMD patients: the LUTEGA study. Graefes Arch Clin Exp Ophthalmol [Internet]. 2013;251(12):2711–23. Available from: https://www.ncbi.nlm.nih.gov/pubmed/23695657

32. García-Layana A, Recalde S, Alamán AS, Robredo PF. Effects of lutein and docosahexaenoic Acid supplementation on macular pigment optical density in a randomized controlled trial. Nutrients [Internet]. 2013;5(2):543–51. Available from: https://www.ncbi.nlm.nih.gov/pubmed/23434908

33. Guymer RH, Baird PN, Varsamidis M, Busija L, Dimitrov PN, Aung KZ, et al. Proof of concept, randomized, placebo-controlled study of the effect of simvastatin on the course of age-related macular degeneration. PLoS One [Internet]. 2013;8(12):e83759. Available from: https://www.ncbi.nlm.nih.gov/pubmed/24391822

34. Huang YM, Yan SF, Ma L, Zou ZY, Xu XR, Dou HL, et al. Serum and macular responses to multiple xanthophyll supplements in patients with early age-related macular degeneration. Nutrition. 2013;29(2):387–92.

35. Jackson TL, Chakravarthy U, Kaiser PK, Slakter JS, Jan E, Bandello F, et al. Stereotactic radiotherapy for neovascular age-related macular degeneration: 52-week safety and efficacy results of the INTREPID study. Ophthalmology [Internet]. 2013;120(9):1893–900. Available from: https://www.ncbi.nlm.nih.gov/pubmed/23490327

36. Krebs I, Schmetterer L, Boltz A, Told R, Vécsei-Marlovits V, Egger S, et al. A randomised double-masked trial comparing the visual outcome after treatment with ranibizumab or bevacizumab in patients with neovascular age-related macular degeneration. Br J Ophthalmol [Internet]. 2013;97(3):266–71. Available from: https://www.ncbi.nlm.nih.gov/pubmed/23292928

37. Krebs I, Vécsei Marlovits V, Bodenstorfer J, Glittenberg C, Ansari Shahrezaei S, Ristl R, et al. Comparison of Ranibizumab monotherapy versus combination of Ranibizumab with photodynamic therapy with neovascular age-related macular degeneration. Acta Ophthalmol [Internet]. 2013;91(3):e178-83. Available from: https://www.ncbi.nlm.nih.gov/pubmed/23241227

38. Menon G, Chandran M, Sivaprasad S, Chavan R, Narendran N, Yang Y. Is it necessary to use three mandatory loading doses when commencing therapy for neovascular age-related macular degeneration using bevacizumab? (BeMOc Trial). Eye (Lond) [Internet]. 2013;27(8):959–63. Available from: https://www.ncbi.nlm.nih.gov/pubmed/23743535

39. Murray IJ, Makridaki M, van der Veen RLP, Carden D, Parry NRA, Berendschot TTJM. Lutein supplementation over a one-year period in early AMD might have a mild beneficial effect on visual acuity: the CLEAR study. Invest Ophthalmol Vis Sci [Internet]. 2013;54(3):1781–8. Available from: https://www.ncbi.nlm.nih.gov/pubmed/23385792

40. Rovner BW, Casten RJ, Hegel MT, Massof RW, Leiby BE, Ho AC, et al. Improving function in age-related macular degeneration: a randomized clinical trial. Ophthalmology [Internet]. 2013;120(8):1649–55. Available from: https://www.ncbi.nlm.nih.gov/pubmed/23642378

41. Russo A, Costagliola C, Delcassi L, Romano MR, Semeraro F. A randomised controlled trial of ranibizumab with and without ketorolac eyedrops for exudative age-related macular degeneration. Br J Ophthalmol [Internet]. 2013;97(10):1273–6. Available from: https://www.ncbi.nlm.nih.gov/pubmed/23873901

42. Souied EH, Delcourt C, Querques G, Bassols A, Merle B, Zourdani A, et al. Oral docosahexaenoic acid in the prevention of exudative age-related macular degeneration: the Nutritional AMD Treatment 2 study. Ophthalmology [Internet]. 2013;120(8):1619–31. Available from: https://www.ncbi.nlm.nih.gov/pubmed/23395546

43. Vingolo EM, Salvatore S, Limoli PG. MP-1 biofeedback: luminous pattern stimulus versus acoustic biofeedback in age related macular degeneration (AMD). Appl Psychophysiol Biofeedback [Internet]. 2013;38(1):11–6. Available from: https://www.ncbi.nlm.nih.gov/pubmed/22903517

44. Chew EY, Clemons TE, Bressler SB, Elman MJ, Danis RP, Domalpally A, et al. Randomized trial of a home monitoring system for early detection of choroidal neovascularization home monitoring of the Eye (HOME) study. Ophthalmology [Internet]. 2014;121(2):535–44. Available from: https://www.ncbi.nlm.nih.gov/pubmed/24211172

45. Bittner AK, Torr-Brown S, Arnold E, Nwankwo A, Beaton P, Rampat R. Improved Adherence to Vision Self-monitoring with the Vision and Memory Stimulating (VMS) Journal for Non-neovascular Age-related Macular Degeneration during a Randomized Controlled Trial. J Clin Exp Ophthalmol. 2014;05(01):1–7.

46. Piri N, Ahmadieh H, Taei R, Soheilian M, Karkhaneh R, Lashay A, et al. Photodynamic therapy and intravitreal bevacizumab with versus without triamcinolone for neovascular age-related macular degeneration; a randomized clinical trial. J Ophthalmic Vis Res. 2014;9(4):469–77.

47. Rezende FA, Lapalme E, Qian CX, Smith LE, SanGiovanni JP, Sapieha P. Omega-3 supplementation combined with anti-vascular endothelial growth factor lowers vitreal levels of vascular endothelial growth factor in wet age-related macular degeneration. Am J Ophthalmol [Internet]. 2014;158(5):1071–8. Available from: https://www.ncbi.nlm.nih.gov/pubmed/25089351

48. Rovner BW, Casten RJ, Hegel MT, Massof RW, Leiby BE, Ho AC, et al. Low vision depression prevention trial in age-related macular degeneration: a randomized clinical trial. Ophthalmology [Internet]. 2014;121(11):2204–11. Available from: https://www.ncbi.nlm.nih.gov/pubmed/25016366

49. Sabour-Pickett S, Beatty S, Connolly E, Loughman J, Stack J, Howard A, et al. Supplementation with three different macular carotenoid formulations in patients with early age-related macular degeneration. Retina [Internet]. 2014;34(9):1757–66. Available from: https://www.ncbi.nlm.nih.gov/pubmed/24887490

50. Scholler A, Richter-Mueksch S, Weingessel B, Vécsei-Marlovits P-V. Differences of frequency in administration of ranibizumab and bevacizumab in patients with neovascular AMD. Wien Klin Wochenschr [Internet]. 2014;126(11):355–9. Available from: https://www.ncbi.nlm.nih.gov/pubmed/24696051

51. Schramm K, Mueller M, Koch FH, Singh P, Kohnen T, Koss MJ. Effects of core vitrectomy in the treatment of age-related macular degeneration. Acta Ophthalmol [Internet]. 2014;92(5):465–72. Available from: https://www.ncbi.nlm.nih.gov/pubmed/24690440

52. Selim A, Koçak N, Aslankara H, Kaynak S. Comparative study of photodynamic therapy monotherapy versus triple management in age-related macular degeneration. Turkish J Med Sci. 2014;44(5):889–95.

53. Sin M, Chrapek O, Karhanova M, Pracharova Z, Langova K, Rehak J. Progression of macular atrophy after PDT combined with the COX-2 inhibitor Nabumetone in the treatment of neovascular ARMD. Biomed Pap Med Fac Univ Palacky Olomouc Czech Repub [Internet]. 2014;158(1):138–43. Available from: https://www.ncbi.nlm.nih.gov/pubmed/23132511

54. Sizmaz S, Kucukerdonmez C, Kal A, Pinarci EY, Canan H, Yilmaz G. Retinal and choroidal thickness changes after single anti-VEGF injection in neovascular age-related macular degeneration: ranibizumab vs bevacizumab. Eur J Ophthalmol [Internet]. 2014;24(6):904–10. Available from: https://www.ncbi.nlm.nih.gov/pubmed/24803153

55. Barikian A, Mahfoud Z, Abdulaal M, Safar A, Bashshur ZF. Induction with intravitreal bevacizumab every two weeks in the management of neovascular age-related macular degeneration. Am J Ophthalmol [Internet]. 2015;159(1):131–7. Available from: https://www.ncbi.nlm.nih.gov/pubmed/25308787

56. Huang YM, Dou HL, Huang FF, Xu XR, Zou ZY, Lu XR, et al. Changes following supplementation with lutein and zeaxanthin in retinal function in eyes with early age-related macular degeneration: A randomised, double-blind, placebo-controlled trial. Br J Ophthalmol. 2015;99(3):371–5.

57. Datseris I, Kontadakis GA, Diamanti R, Datseris I, Pallikaris IG, Theodossiadis P, et al. Prospective comparison of low-fluence photodynamic therapy combined with intravitreal bevacizumab versus bevacizumab monotherapy for choroidal neovascularization in age-related macular degeneration. Semin Ophthalmol [Internet]. 2015;30(2):112–7. Available from: https://www.ncbi.nlm.nih.gov/pubmed/24117412

58. Hatz K, Schneider U, Henrich PB, Braun B, Sacu S, Prünte C. Ranibizumab plus verteporfin photodynamic therapy in neovascular age-related macular degeneration: 12 months of retreatment and vision outcomes from a randomized study. Ophthalmologica [Internet]. 2015;233(2):66–73. Available from: https://www.ncbi.nlm.nih.gov/pubmed/25471330

59. Kuppermann BD, Goldstein M, Maturi RK, Pollack A, Singer M, Tufail A, et al. Dexamethasone Intravitreal Implant as Adjunctive Therapy to Ranibizumab in Neovascular Age-Related Macular Degeneration: A Multicenter Randomized Controlled Trial. Ophthalmologica. 2015;234(1):40–54.

60. Li B, Powell A-M, Hooper PL, Sheidow TG. Prospective evaluation of teleophthalmology in screening and recurrence monitoring of neovascular age-related macular degeneration: a randomized clinical trial. JAMA Ophthalmol [Internet]. 2015;133(3):276–82. Available from: https://www.ncbi.nlm.nih.gov/pubmed/25473945

61. Markun S, Dishy A, Neuner-Jehle S, Rosemann T, Frei A. The Chronic Care for Wet Age Related Macular Degeneration (CHARMED) Study: A Randomized Controlled Trial. PLoS One [Internet]. 2015;10(11):e0143085. Available from: https://www.ncbi.nlm.nih.gov/pubmed/26569501

62. Semeraro F, Russo A, Delcassi L, Romano MR, Rinaldi M, Chiosi F, et al. TREATMENT OF EXUDATIVE AGE-RELATED MACULAR DEGENERATION WITH RANIBIZUMAB COMBINED WITH KETOROLAC EYEDROPS OR PHOTODYNAMIC THERAPY. Retina [Internet]. 2015;35(8):1547–54. Available from: https://www.ncbi.nlm.nih.gov/pubmed/25784358

63. Wolf-Schnurrbusch UEK, Zinkernagel MS, Munk MR, Ebneter A, Wolf S. Oral Lutein Supplementation Enhances Macular Pigment Density and Contrast Sensitivity but Not in Combination With Polyunsaturated Fatty Acids. Invest Ophthalmol Vis Sci [Internet]. 2015;56(13):8069–74. Available from: https://www.ncbi.nlm.nih.gov/pubmed/26720458

64. Zehetner C, Kralinger MT, Modi YS, Waltl I, Ulmer H, Kirchmair R, et al. Systemic levels of vascular endothelial growth factor before and after intravitreal injection of aflibercept or ranibizumab in patients with age-related macular degeneration: a randomised, prospective trial. Acta Ophthalmol [Internet]. 2015;93(2):e154-9. Available from: https://www.ncbi.nlm.nih.gov/pubmed/25488124

65. Lashay A, Sadough G, Ashrafi E, Lashay M, Movassat M, Akhondzadeh S. Short-term Outcomes of Saffron Supplementation in Patients with Age-related Macular Degeneration: A Double-blind, Placebo-controlled, Randomized Trial. Med hypothesis, Discov Innov Ophthalmol J [Internet]. 2016;5(1):32–8. Available from: http://www.ncbi.nlm.nih.gov/pubmed/28289690%0Ahttp://www.pubmedcentral.nih.gov/articlerender.fcgi?artid=PMC5342880

66. Rezar-Dreindl S, ra, Sacu S, Eibenberger K, Pollreisz A, Bühl W, et al. The Intraocular Cytokine Profile and Therapeutic Response in Persistent Neovascular Age-Related Macular Degeneration. f [Internet]. 2016;57(10):4144–50. Available from: https://www.ncbi.nlm.nih.gov/pubmed/27537264

67. Saviano S, ro, Leon PE, Mangogna A, ro, Tognetto D. Combined therapy (intravitreal bevacizumab plus verteporfin photodynamic therapy) versus intravitreal bevacizumab monotherapy for choroidal neovascularization due to age-related macular degeneration: a 1-year follow-up study. Digit J Ophthalmol DJO [Internet]. 2016;22(2):46–53. Available from: https://www.ncbi.nlm.nih.gov/pubmed/27582675

68. Tao Y, Jiang P, Wei Y, Wang P, Sun X, Wang H. α-Lipoic Acid Treatment Improves Vision-Related Quality of Life in Patients with Dry Age-Related Macular Degeneration. Tohoku J Exp Med [Internet]. 2016;240(3):209–14. Available from: https://www.ncbi.nlm.nih.gov/pubmed/27840374

69. Weingessel B, Mihaltz K, Vécsei-Marlovits PV. Predictors of 1-year visual outcome in OCT analysis comparing ranibizumab monotherapy versus combination therapy with PDT in exsudative age-related macular degeneration. Wien Klin Wochenschr [Internet]. 2016 Aug;128(15):560–5. Available from: https://www.ncbi.nlm.nih.gov/pubmed/25787216

70. Dong Y, Wan G, Yan P, Chen Y, Wang W, Peng G. Effect of anti-VEGF drugs combined with photodynamic therapy in the treatment of age-related macular degeneration. Exp Ther Med [Internet]. 2016 Dec;12(6):3923–6. Available from: https://www.ncbi.nlm.nih.gov/pubmed/28105123

71. Mantel I, Gianniou C, Dirani A. CONVERSION TO AFLIBERCEPT THERAPY VERSUS CONTINUING WITH RANIBIZUMAB THERAPY FOR NEOVASCULAR AGE-RELATED MACULAR DEGENERATION DEPENDENT ON MONTHLY RANIBIZUMAB TREATMENT. Retina [Internet]. 2016;36(1):53–8. Available from: https://www.ncbi.nlm.nih.gov/pubmed/26166797

72. Abdelfattah NS, Al-Sheikh M, Pitetta S, Mousa A, Sadda SR, Wykoff CC, et al. Macular Atrophy in Neovascular Age-Related Macular Degeneration with Monthly versus Treat-and-Extend Ranibizumab: Findings from the TREX-AMD Trial. Ophthalmology [Internet]. 2017;124(2):215–23. Available from: https://www.ncbi.nlm.nih.gov/pubmed/27863845

73. Akuffo KO, Nolan JM, Howard AN, Moran R, Stack J, Klein R, et al. Sustained supplementation and monitored response with differing carotenoid formulations in early age-related macular degeneration. Eye (Lond) [Internet]. 2015;29(7):902–12. Available from: https://www.ncbi.nlm.nih.gov/pubmed/25976647

74. Mori R, Tanaka K, Haruyama M, Kawamura A, Furuya K, Yuzawa M. Comparison of pro re nata versus Bimonthly Injection of Intravitreal Aflibercept for Typical Neovascular Age-Related Macular Degeneration. Ophthalmologica [Internet]. 2017;238(1):17–22. Available from: https://www.ncbi.nlm.nih.gov/pubmed/28402983

75. Sengul A, Rasier R, Ciftci C, Artunay O, Kockar A, Bahcecioglu H, et al. Short-term effects of intravitreal ranibizumab and bevacizumab administration on 24-h ambulatory blood pressure monitoring recordings in normotensive patients with age-related macular degeneration. Eye (Lond) [Internet]. 2017 May;31(5):677–83. Available from: https://www.ncbi.nlm.nih.gov/pubmed/28060360

76. Azar G, Maftouhi Q, Masella J, Mauget-Faysse M. Macular pigment density variation after supplementation of lutein and zeaxanthin using the Visucam® 200 pigment module: Impact of age-related macular degeneration and lens status. J Fr Ophtalmol [Internet]. 2017;40(4):303–13. Available from: https://www.ncbi.nlm.nih.gov/pubmed/28336284

77. Figurska M, Bogdan-B, urska A, Rękas M. Effect of Phacoemulsification on Visual Acuity and Macular Morphology in Patients with Wet Age-Related Macular Degeneration. Med Sci Monit [Internet]. 2018;24:6517–24. Available from: https://www.ncbi.nlm.nih.gov/pubmed/30220702

78. Li S, Liu N, Lin L, Sun E-D, Li J-D, Li P-K. Macular pigment and serum zeaxanthin levels with Goji berry supplement in early age-related macular degeneration. Int J Ophthalmol [Internet]. 2018;11(6):970–5. Available from: https://www.ncbi.nlm.nih.gov/pubmed/29977809

79. Motarjemizadeh Q, Aidenloo NS, Abbaszadeh M, Sadrinia V. Intravitreal Bevacizumab with or without Triamcinolone for Wet Age-related Macular Degeneration: Twelve-month Results of a Prospective, Randomized Investigation. Middle East Afr J Ophthalmol [Internet]. 2018 Jan;25(1):1–7. Available from: https://www.ncbi.nlm.nih.gov/pubmed/29899643

80. Rosenfeld PJ, Dugel PU, Holz FG, Heier JS, Pearlman JA, Novack RL, et al. Emixustat Hydrochloride for Geographic Atrophy Secondary to Age-Related Macular Degeneration: A Randomized Clinical Trial. [Internet]. Vol. 125, Ophthalmology. 2018. p. 1556–67. Available from: https://www.ncbi.nlm.nih.gov/pubmed/29716784

81. Russo A, Scaroni N, Gambicorti E, Turano R, Morescalchi F, Costagliola C, et al. Combination of ranibizumab and indomethacin for neovascular age-related macular degeneration: randomized controlled trial. Clin Ophthalmol [Internet]. 2018;12:587–91. Available from: https://www.ncbi.nlm.nih.gov/pubmed/29628756

82. Giancipoli E, Pinna A, Boscia F, Zasa G, Sotgiu G, Dore S, et al. Intravitreal Dexamethasone in Patients with Wet Age-Related Macular Degeneration Resistant to Anti-VEGF: A Prospective Pilot Study. Available from: file:///pubmed/30151278

83. Broadhead GK, Grigg JR, McCluskey P, Hong T, Schlub TE, Chang AA. Saffron therapy for the treatment of mild/moderate age-related macular degeneration: a randomised clinical trial. Graefes Arch Clin Exp Ophthalmol [Internet]. 2019 Jan;257(1):31–40. Available from: https://www.ncbi.nlm.nih.gov/pubmed/30343354

84. Guymer RH, Wu Z, Hodgson LAB, Caruso E, Brassington KH, Tindill N, et al. Subthreshold Nanosecond Laser Intervention in Age-Related Macular Degeneration: The LEAD Randomized Controlled Clinical Trial. Ophthalmology [Internet]. 2019;126(6):829–38. Available from: https://www.ncbi.nlm.nih.gov/pubmed/30244144

85. Kaltenegger K, Kuester S, Altpeter-Ott E, Eschweiler GW, Cordey A, Ivanov I V, et al. Effects of home reading training on reading and quality of life in AMD-a randomized and controlled study. Graefes Arch Clin Exp Ophthalmol [Internet]. 2019 Jul;257(7):1499–512. Available from: https://www.ncbi.nlm.nih.gov/pubmed/31111250

86. Liu K, Song Y, Xu G, Ye J, Wu Z, Liu X, et al. Conbercept for Treatment of Neovascular Age-related Macular Degeneration: Results of the Randomized Phase 3 PHOENIX Study. Am J Ophthalmol. 2019;197:156–67.

87. Luo D, Deng T, Yuan W, Deng H, Meng H, Jin M. Effects of Huangban Bianxing One decoction combined with ranibizumab on treating exudative age-related macular degeneration. J Tradit Chinese Med = Chung i tsa chih ying wen pan. 2019;39(6):892–901.

88. Markowitz SN, Devenyi RG, Munk MR, Croissant CL, Tedford SE, Rückert R, et al. Study With Photobiomodulation for the Treatment of Dry Age- Related Macular Degeneration. Retina. 2019;

89. Nunes RP, Hirai FE, Barroso LF, es, Badaró E, Novais E, et al. Effectiveness of monthly and fortnightly anti-VEGF treatments for age-related macular degeneration. Arq Bras Oftalmol [Internet]. 2019 May;82(3):225–32. Available from: https://www.ncbi.nlm.nih.gov/pubmed/30810619

90. Parravano M, Tedeschi M, Manca D, Costanzo E, Di Renzo A, Giorno P, et al. Effects of Macuprev® Supplementation in Age-Related Macular Degeneration: A Double-Blind Randomized Morpho-Functional Study Along 6 Months of Follow-Up. Adv Ther [Internet]. 2019;36(9):2493–505. Available from: https://www.ncbi.nlm.nih.gov/pubmed/31243641

91. Semeraro F, Gambicordi E, Cancarini A, Morescalchi F, Costagliola C, Russo A. Treatment of exudative age-related macular degeneration with aflibercept combined with pranoprofen eye drops or nutraceutical support with omega-3: A randomized trial. Br J Clin Pharmacol [Internet]. 2019;85(5):908–13. Available from: https://www.ncbi.nlm.nih.gov/pubmed/30680768

92. Yuan J. Role of inflammatory factors in the effects of aflibercept or ranibizumab treatment for alleviating wet age-associated macular degeneration. Exp Ther Med [Internet]. 2019 May;17(5):4249–58. Available from: https://www.ncbi.nlm.nih.gov/pubmed/30988797

93. Gillies MC, Hunyor AP, Arnold JJ, Guymer RH, Wolf S, Pecheur FL, et al. Macular Atrophy in Neovascular Age-Related Macular Degeneration: A Randomized Clinical Trial Comparing Ranibizumab and Aflibercept (RIVAL Study). Ophthalmology [Internet]. 2019;127(2):198–210. Available from: https://doi.org/10.1016/j.ophtha.2019.08.023

94. Hsu J, Patel SN, Wolfe JD, Shah CP, Chen E, Jenkins TL, et al. Effect of Adjuvant Topical Dorzolamide-Timolol vs Placebo in Neovascular Age-Related Macular Degeneration: A Randomized Clinical Trial. JAMA Ophthalmol [Internet]. 2020; Available from: https://www.ncbi.nlm.nih.gov/pubmed/32239190

95. Piatti A, Croce A, Mazzacane D, Traina G, Ambrosino L, Boni L, et al. Effect of 2-year nutritional supplementation on progression of age-related macular degeneration. Eur J Ophthalmol. 2020;30(2):376–81.

96. Sawa M, Shunto T, Nishiyama I, Yokoyama A, Shigeta R, Miura S, et al. Effects of Lutein Supplementation in Japanese Patients with Unilateral Age-Related Macular Degeneration: The Sakai Lutein Study. Sci Rep [Internet]. 2020;10(1):5958. Available from: https://www.ncbi.nlm.nih.gov/pubmed/32249850
