# Supplementary material for: Linguistic spin in randomized controlled trials about age-related macular degeneration
Source: Front Epidemiol. 2022 Oct 31;2:961996. doi: 10.3389/fepid.2022.961996 (PMC10910936; doi:10.3389/fepid.2022.961996)
Supplement: Supplementary file 4 [file Table_4.DOCX]

**Supplementary Table D. Linguistic spin examples defined by category identified in the RCTs included**

| Study ID^*^ | Abstract  *Category* | *Example (number)* | Main text  *Category* | *Example (number)* |
| --- | --- | --- | --- | --- |
| 1 | 4 | Significant predictors | 1  3  4 | Numerically larger; but our study may have been underpowered to detect a small difference  Trend for a higher  Statistically significant |
| 2 | 4  5 | Significantly increases  (2x) | 1  2  3 | A borderline significantly greater extent  Statistically similar  Greatly exceeds (3x), good compliance |
| 3 | 3  7 | Improved markedly, rapid improvement  First report, results that seem to justify further research | 3  7 | Improved markedly, rapid improvement, increased markedly, positive trends, encouraging trends, beneficial effect, positive feature, we see potential in  Our report is the first |
| 4 | 5 | (1x) | 2  3  7 | Virtually identical  Remained superior, making the results of our study unique  This is the first time |
| 5 | 4 | Significantly higher | 3  4 | The present study stands out  Increased significantly (2x), ‘elevated the xanthophyll…in plasma significantly’, significantly higher |
| 6 | - | - | - | - |
| 7 | 3  4 | Trend for reduction, trend for reduced, are encouraging, potentially beneficial effect  Statistically significant | 3  4  7 | A trend for, trend for a reduction  Statistically significant  The first study, is the first |
| 8 | 4 | Statistically significant | 3  4 | Highly statistically significant  Statistically significant (3x) |
| 9 | 3  4 | Positive results  Significantly higher | 3  4 | Trend to be (2x), positive results  Significant reduction, significantly higher |
| 10 | - | - | 7 | Even a benefit like that could be very important; was important at least for the quality |
| 11 | 3 | May produce greater benefits | 3  4  7 | Trend toward, additive response  Significantly more variable, statistically but not clinically significant  Suggesting that |
| 12 | 4 | Significant increases (2x) | 1  2  3  4 | Correlated positively  Tendency for a treatment effect  Tendency for a treatment effect, tendency toward improvement (3x)  Significant increase (2x), significant within-group increase, significant MPOD increase, most significant difference, significant association |
| 13 | 3 | A trend favoring, showed superiority | 3  4 | This trend (2x)  ﻿Statistically significant |
| 14 | 4 | Significantly reduced | 3  4 | Promising results  Significantly less than, significant mean improvement, significant improvement, significant reduction, significantly lower |
| 15 | 3  5 | Considerable increase  (1x) | 3  4 | Tended to result, trend to improvement, trend toward improvement, considerable increase, highly significant, very fast significant increase  Significant enrichment (2x), significant decrease (2x), significant increase (2x), significant improvement |
| 16 | - | - | - | - |
| 17 | 4 | Significant improvement, significantly improves | 1  3  4 | ‘The observed CRT … and retinal atrophy’  This tendency, the first  Significant BCVA improvement, significant improvement, significantly higher, significantly improves |
| 18 | 6 | (1x) | 3  4 | Very significantly, beneficial effect  Significant increase, significantly improve |
| 19 | - | - | 7 | The first study |
| 20 | - | - | 3 | ‘Nonsignificant trends, the tendency’ |
| 21 | 1 | Tended to decrease | 1  2  7 | Tended to be greater (2x)  ‘Although this difference…meaningful to reduce’  Which further supports the efficacy of |
| 22 | 3 | Most prominent effect | 1  3  7 | Marginally significant; ‘although theoretically this… was still found’  Tendency towards, large reduction, highly significant, the overall trend is for, strongly indicate  The first |
| 23 | 3  7 | Compelling evidence  May have a role in | 1  3  7 | ﻿Direction of the treatment effect was for a reduction in  Strong evidence, highly clinically meaningful, potential beneficial effect  The first, crucial information |
| 24 | - | - | 3 | Greater reduction |
| 25 | - | - | - | - |
| 26 | 4 | Significantly increased | 3  4  7 | Tended to show, strenghtened our results, positively responsive, strong proof  Significantly increased, significantly higher, most significant, significant increase, increased significantly  Responded positively, notable changes, this is the first |
| 27 | 3  4 | Favorable safety profile  Significantly reduces | 3 | Encouraging structural and functional outcomes |
| 28 | 4 | Improved significantly (2x), significant  difference | 3  4  7 | Highly significant (2x), benefited considerably, highly significantly, clearly benefited  Statistically significant (3x), increased significantly (2x), highly significant (2x), significant improvement (3x), improved highly significantly, significant difference (2x)  Is beneficial |
| 29 | - | - | 2  3  7 | ﻿Equivalent to ranibizumab  Tended to be higher, tended to be more prominent, a tendency towards  ﻿There was a steep increase |
| 30 | 4 | Decreased significantly, significant reduction | 3  4  6 | Favourable results  Significant larger, significant result, significant reduction, significantly reduced (2x)  ﻿Significantly reduced in both groups (although not significant) |
| 31 | 4  5  7 | Significantly different  (1x)  Acceptable tolerability | 3  4  7 | ﻿Clear decrease, responded favorably to, beneficial effect Significantly delayed, significantly reduced, statistiscally significant (2x), clinically significant  ﻿Has the potential to influence |
| 32 | 4  5 | Significant improvement  (1x) | 1  4  7 | ﻿These changes could not be proven statistically due to the small sample size  Significantly increased, s﻿ignificant changes in, statistically significant, significant improvement  Greater benefit |
| 33 | - | - | 7 | This is the first study |
| 34 | 3 | May be an effective therapeutic intervention | 3  4  7 | ﻿Important finding, may have some benefits for  ﻿Statistically significant improvement  ﻿We are the first to report |
| 35 | 7 | Clinical benefit | 3  4  7 | Clinical benefit  ﻿Significant and rapid improvement, statistically significant  ﻿Has the potential to be, promising results, this is the first report, highlight the importance of the findings |
| 36 | 7 | Tended to favor | 3 | ﻿Substantial reduction, trend toward, greater reduction, apparent benefits of, are encouraging |
| 37 | - | - | 4 | Increased significantly, significantly increased, significantly restore, the macular region significantly |
| 38 | 4 | Significant increases | 3  4 | ﻿A much greater increase, tendency for, trend toward decrease  Significant increases, significantly increased, significant associations |
| 39 | 3  4 | Trend toward improvement  ﻿Increased significantly, significant dose-response, significant between-group difference | 1  3  4 | ‘However it should…may be explained’  Progressive improvements, small but appreciable, important finding, important clinical implications, intriguing finding, safe and effective therapy, may have some benefit  Significant dose-response effect, significant increases, significant increase, significant relationships |
| 40 | 3  4 | More robust benefits  Statistically improved | 3  4  7 | Showed a positive trend, a trend was, more robustly seen  ﻿Significant improvements (3x), significant improvement, statistically significant benefit, is significant  ‘None of the AEs…related to the treatment’, illustrate positive benefit, beneficial changes, may be measureable in regards, multiple clinical and anatomical benefits |
| 41 | - | - | 1  3  4  7 | ﻿‘Possibly a longer…the CCM implementation’  Trend of retinal thickness, trend went upwards  ﻿Significant improvements, improved significantly (2x)  The first study |
| 42 | - | - | 1  2  3  7 | ‘This difference was…the small numbers’, ‘post hoc analysis…small expected difference’  ‘Outcomes between the…are moderately good’  Highly significant, tends to give, was small but probably clinically relevant and justifie  This is the first |
| 43 | 4 | Significantly improved, significantly decreased, significant improvement | 1  3  4  7 | ﻿‘The reason for…had already occurred’  Superior to  Significantly decreased  ﻿Showed a trend, this is the first study |
| 44 | 4 | Reduced significantly | 2  3  4 | ﻿Statistically borderline difference  Statistically more pronounced, trend toward greater treatment benefit  Remained significant, significantly higher in, significant benefit |
| 45 | - | - | 3  4  7 | ﻿Strong main effect, highly significant increase, clear link, particularly compelling, clear benefits of  Highly significant increase, significant changes, significant difference  ﻿Study is novel, for the first time |
| 46 | 4 | Statistically significant | 1  3  7 | ﻿Showed a tendency for greater resolution  ﻿Tendency toward (2x), a greater tendency  The first study |
| 47 | - | - | 4 | Significantly decreased (4x) |
| 48 | 4 | Significant increase | 7 | Highlighted, suggesting a functional improvement |
| 49 | - | - | 3  7 | ﻿We evaluate the successful of  For the first time |
| 50 | - | - | 3  4  7 | Significant trend toward, positive effects  Significantly better*,* significant within-group (2x), significant trend toward, significantly improved  ﻿Clinically relevant improvements, powerful biological antioxidant |
| 51 | 3  4 | Trend toward  Statistically significant, significant reduction | 3  4 | Trend of, positive effect on, trend toward, significant functional, trend towards  Were significant, statistically significant reduction |
| 52 | - | - | 1  3  4 | Positive correlation  Showed a distinct  Significantly decreased |
| 53 | - | - | 3  4  6 | Tended toward improvement  Significant decrease, significantly lower (2x)  (1x) |
| 54 | - | - | 4  7 | Significantly shortened, decrease significantly  This is the first (2x) |
| 55 | - | - | 3  4 | Is significant (2x)  Is significant (2x) |
| 56 | - | - | 7 | These findings are important |
| 57 | 3 | Associated with greater improvement | 3  7 | Was strong, remarkably good vision  Is the first |
| 58 | 4  7 | Significant improvement  Is the first | 4  7 | Significant reduction, significantly greater reduction  Is the first |
| 59 | 3  4 | Very beneficial, provides superior anatomical and visual outcomes  Significant improvement | 3  4 | Has the potential to, greatly beneficial  Improved significantly, significant reduction (2x), significant BCVA improvement |
| 60 | - | - | 3  4 | Are rendered all the more clinically meaningful  Significantly greater, significant rise |
| 61 | 4 | Significant reduction | 3 | Is remarkable, tends to plateau, findings are promising, appears to be a useful therapeutic choice |
| 62 | 1  4 | Marginally significant  Significant increase | 1  3  4 | Marginally significant, ‘the lack of…in this study’  Tendency toward, increasing tendency  Significant increase, significantly increased |
| 63 | - | - | - | - |
| 64 | - | - | 3 | A trend towards |
| 65 | 4 | Significantly reduced | 3  4 | Marked decrease  Statistically significant |
| 66 | - | - | 4 | Significantly improved |
| 67 | 4 | Significant improvement | 3  4 | Improved quickly, valuable therapeutic support  Significant increase, slight but significant difference |
| 68 | 4 | Significant improvement | 3  4  7 | Have the potential to, has the potential to  Significant improvement, significant reduction, slightly significant BCVA, significant BCVA  This is the first |
| 69 | - | - | 3 | Our findings emphasize the beneficial role |
| 70 | 4 | Significant reduction | 4  7 | Significant decrease  The first study |
| 71 | 4 | Significant decrease | 2  4 | Slight but insignificant decrease  Significant decrease (2x) |
| 72 | 4 | Significanty reduced | 3  4  7 | Increased markedly  Increasing significantly, but significantly, significant increase (2x), significant postbaseline increase, significant changes, clinically significant, increased significantly, significant EPA plus DHA increase, significant differences  Is the first |
| 73 | 2  4 | Slight but statistically nonsignificant  Statistically significant | 2  3  4 | Slight but statistically nonsignificant  Trend for a decrease  Statistically significant, significantly increase |
| 74 | 3  4 | Regarding as a promising agent  Statistically significant increase, improved significantly, significantly higher | 4 | Improved significantly, significant increase (2x), significantly improved, can significantly elevate |
| 75 | 4 | Statistically significant | 3  4 | Very impressive  Statistically significant |
| 76 | 3 | Decreased considerably | 3  4  7 | Decreased considerably, a more distinct reduction  Significant reduction  This is the first study |
| 77 | - | - | 3 | Trends toward |
| 78 | 4 | Increased significantly | 3  4 | Highly significant, drastically decreased, may still be beneficial, strikingly  Significantly lower |
| 79 | 3 | Markedly decreased, markedly improved | 3  4 | Significantly higher  Improved significantly, significantly higher, decreased significantly, significant difference was identified, more significant than |
| 80 | 4 | Significantly reduced | 3  4 | Novel finding  Significant reduction (3x) |
| 81 | - | - | 3  4 | Was beneficial  Significantly decreased (2x), significant reduction, was also significant, significant IOP increase |
| 82 | - | - | 3  4 | Highly valuable  Significantly higher improvement, statistically significant (4x) |
| 83 | 4 | Significantly affect | 1  3  4  7 | A nonsignificant tendency  Strong significant stimulating, strong positive stimulating, this tendency, the tendency of  Significantly higher level, significantly higher in older  Surprisingly |
| 84 | 4 | Reduced significantly | 3  4 | Beneficial effect, beneficial effects  Statistically significant reduction, significantly decreased, significant effect (2x), significant influence, significantly more pronounced |
| 85 | - | - | 3  4 | Tended to report, strongly argue in favor of a positive effect of, results were encouraging  Significant treatment effect (2x), significant improvement, for the significant crossover effect |
| 86 | - | - | - | - |
| 87 | - | - | 3 | Trend towards better (2x) |
| 88 | - | - | 1  4  6 | Showed a considerably efficacy  Was significantly less, significant improvement  (1x) |
| 89 | - | - | 4 | Significant improvement |
| 90 | 4 | Statistically significant, significantly reduced | 4 | Significantly reduced (2x) |
| 91 | 4 | Significantly different | 3  4 | Potentially important  Significantly improved, significant effect of, statistically significant differences |
| 92 | 3 | Tendency toward an increase | 1  3  4  7 | But a tendency toward an improvement, a tendency toward an increase was, tendency toward improvement  Highly significant, clearly reveals  Significant association, significant correlation  Was the first |
| 93 | 2  3 | Trend toward significance  Distinct visual benefits | 1  3  4  7 | Trend for improvement, near improvement, a trend in  Remarkable increased visual acuity  Statistically significant, significantly elevated  Has unique |
| 94 | 3  4 | Substantial reduction  Significantly higher | 3  4 | Great rheological effectiveness, important sign, our results highlight positive therapeutic effects  Significantly lower (2x), improve significantly, statistically significant (3x), significantly higher, increased significantly (2x), significantly longer, significant increase, influenced significantly, significantly reduced |
| 95 | - | - | 3  7 | Nearly significant  Our study is the only one that has fulfilled these requirements |
| 96 | 4 | Significant decrease (5x), significant changes | 4 | Increased the BCVA significantly, significant increase, significant decreases, significant decrease |

*^*^ Studies are pseudonymized. Linguistic spin categories: 1. Use of words to reject or explain non-statistically signifant results; 2. Use of words to claim comparable effectiveness or equivalence despite P > .05; 3. Use of words to point out the beneficial effect of the treatment investigated; 4. Use of ‘(statistically) significant/significance’ without reporting a P-value or a 95% CI for results showing a beneficial* *effect of the treatment investigated; 5. Particular focus on results with statistical significance in abstract and or main text; 6.* Inconsistency in the significance reported for the same results within the article*; 7. Other forms of linguistic spin.*
